# GILT stabilizes cofilin to promote the metastasis of prostate cancer

**DOI:** 10.1038/s41420-025-02288-0

**Published:** 2025-01-16

**Authors:** Dunsheng Han, Zhiming Wu, Cong Zhang, Ziwei Wei, Fan Chao, Xuefeng Xie, Jinke Liu, Yufeng Song, Xiaoming Song, Dingchang Shao, Shiyu Wang, Guoxiong Xu, Gang Chen

**Affiliations:** 1 Department of Urology, Jinshan Hospital, Fudan University, Shanghai, China; 2 Department of Urology, Sun Yat-sen University Cancer Center, Guangzhou, China; 3 Department of Urology, Zhongshan Hospital, Fudan University (Xiamen Branch), Xiamen, China; 4 Research Center for Clinical Medicine, Jinshan Hospital, Fudan University, Shanghai, China

**Keywords:** Prostate cancer, Metastasis

## Abstract

Gamma-interferon-induced lysosomal thiol reductase (GILT), known for catalyzing disulfide bond reduction, is involved in various physiological processes. While the involvement of GILT in the development of various tumors has been demonstrated, the mechanisms underlying its regulation in prostate cancer (PCa) are not fully understood. In the present study, we confirmed that GILT was significantly upregulated in PCa and facilitated tumor metastasis. Mechanistically, GILT stabilized the cofilin protein by competitively binding to cofilin with Src family tyrosine kinase (SRC), inhibiting SRC-mediated tyrosine phosphorylation of cofilin, thereby suppressing the ubiquitination pathway degradation of cofilin. GILT overexpression stabilized and increased the protein level of cofilin in PCa cells and promoted the metastasis of PCa cells by accelerating actin dynamics through cofilin-mediated actin severing. Our findings reveal a novel mechanism of GILT in PCa and provide a new potential target for the diagnosis and treatment of PCa patients.

## Introduction

Prostate cancer (PCa) is one of the most common malignancies in men. In 2023, the American Cancer Society reported 288,300 new cases of PCa, accounting for 29% of male tumors, ranking first in male tumor incidence [1]. Early-stage localized PCa can be effectively treated with radical surgery, radioactive seed implantation, or external beam radiotherapy. However, up to 54% of newly diagnosed cases in China present with metastatic prostate cancer (mPCa) [2], and the vast majority of these patients experience a relapse, progressing to castration-resistant prostate cancer (CRPC) [3], Therefore, uncovering the molecular mechanisms underlying the metastasis and progression of PCa is of great significance.

Gamma-interferon-inducible lysosomal thiol reductase (GILT) is the only known enzyme that catalyzes disulfide bond reduction in the endocytic pathway [4, 5]. GILT boosts major histocompatibility complex (MHC) class II antigen processing for CD4^+^ T cell activation and immune regulation [6], while also reducing oxidative stress and autophagy [7, 8]. Recently, as more studies have detected abnormal GILT expression in various cancers, some functions of GILT in cancer progression have been reported [7, 9, 10]. For instance, researchers co-cultured monocytes with lung adenocarcinoma cells to mimic the tumor microenvironment, subsequently observing an upregulation of GILT expression in the lung adenocarcinoma cells, which was correlated with enhanced cancer cell migration [11]. A previous study demonstrated that intergrating the expression levels of GILT with those of other lysosomal genes, alongside the Gleason score, enhances the prediction of PCa prognosis [12]. Furthermore, the genetic mutations of GILT are significantly related to the prognosis of androgen deprivation therapy (ADT) [13]. Despite these findings, the mechanisms by which GILT influences human PCa remain elusive.

Cofilin is a key regulator of actin dynamics and plays an essential role in cell spreading and migration [14]. It facilitates recycling by disassembling filamentous actin (F-actin) into globular monomeric actin (G-actin) within the actin network, thereby preparing for subsequent actin polymerization at the leading edge of cells [15]. Additionally, cofilin’s severing of actin filaments creates free barbed ends that serve as sites for actin polymerization and nucleation of new filaments [16, 17]. This is critical for membrane protrusion at the cell front during movement. The Src family tyrosine kinase (SRC) plays a role in various signaling pathways [18, 19]. Its phosphorylation activity is crucial for regulating cell adhesion, spreading, migration, and invasion [20, 21]. Protein phosphorylation is known to act as a marker, targeting modified proteins to the ubiquitination-proteosome pathway [22].

In this study, for the first time, we identified a novel role for GILT in promoting PCa metastasis and explored its underlying regulatory mechanisms. Our findings reveal a potential therapeutic target for PCa treatment.

## Results

### GILT is overexpressed in PCa and is associated with a poor prognosis

**Fig. 1:**
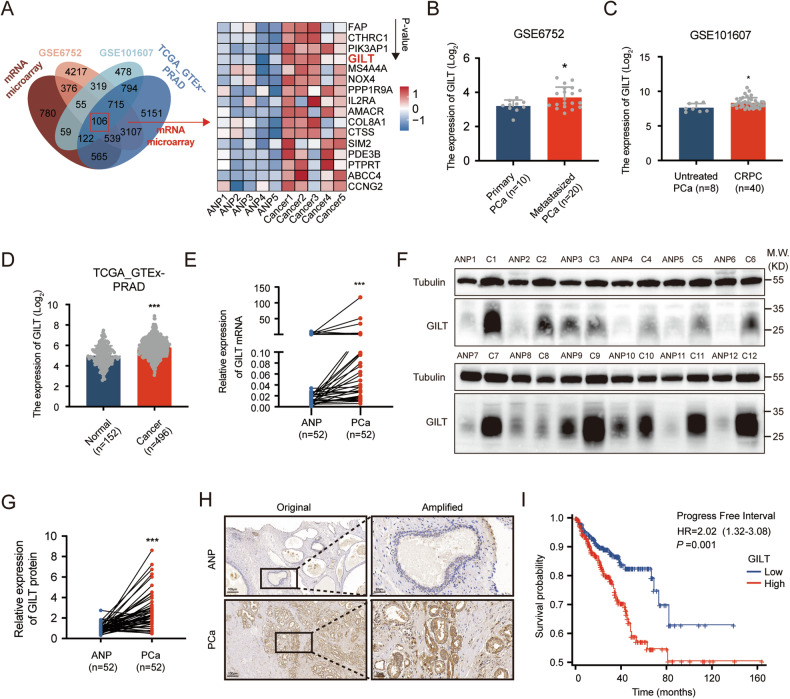
GILT is overexpressed in PCa and is correlated with poor prognosis. **A** Venn diagrams (left) demonstrate the significant upregulation of mRNA overlap across four PCa datasets, including PCa samples microarray, GSE6752, GSE101607, and TCGA_GTEx-PRAD datasets. Heatmap results (right) display the expression levels of the 106 genes featured in the microarray result, sorted by *p*-value. **B** GILT is higher expressed in metastatic PCa tissues than in primary PCa tissues (GSE6752). *p < 0.05, Welch’s *t*-test. **C** GILT is higher expressed in CRPC tissues than in untreated PCa tissues (GSE101607). *p < 0.05, Wilcoxon rank sum test. **D** GILT is higher in PCa tissues than in normal tissues (TCGA database). ***p < 0.001, Student’s *t*-test. **E** GILT mRNA is upregulated in PCa tissues as compared with adjacent non-cancerous prostate (ANP) tissues by RT-qPCR (*n* = 52). ***p < 0.001, Wilcoxon signed-rank test. **F**, **G** The protein level of GILT is higher in fresh PCa tissues than in paired ANP tissues (*n* = 52). ***p < 0.001, Wilcoxon signed-rank test. **H** Immunohistochemical results reveal that GILT is mainly distributed in the cytoplasm of PCa cells and is highly expressed in PCa tissues (scale bar, 100 μm or 20 μm). **I** Kaplan–Meier curve shows the prognostic value of GILT in PFI of PCa patients. ***p < 0.001, log-rank test. GILT gamma-interferon-inducible lysosomal thiol reductase, PCa prostate cancer, TCGA The Cancer Genome Atlas Program, GTEx Genotype-Tissue Expression, RT-qPCR reverse transcription-quantitative polymerase chain reaction, ANP adjacent non-cancerous prostate, GSE Gene Expression Omnibus Series, PRAD prostate adenocarcinoma, CRPC castration-resistant prostate cancer, PFI progression-free interval.

To investigate the underlying mechanisms of PCa, we employed microarray analysis on five pairs of fresh PCa samples. To gain further insight into the malignant progression of PCa, we incorporated published data on PCa metastasis (GSE6752) [23], androgen resistance (GSE101607) [24], and the TCGA_GTEx-PRAD dataset. By cross-analyzing genes that were significantly overexpressed across the four datasets (fold change ≥1.5, *p* < 0.05), we identified 106 genes that showed consistent overexpression. We then ranked these genes based on their *p*-values in our microarray data (Fig. 1A). The aberrant GILT overexpression among these genes garnered particular attention (Fig. 1B–D). Collectively, these data suggest that GILT may play a role in the clinical progression of PCa, which warrants further attention. For further validation of these findings, an additional 52 pairs of PCa and ANP tissue samples were collected. GILT overexpression in PCa tissues was corroborated using RT-qPCR and Western blot analyses (Fig. 1E–G) and further substantiated by immunohistochemistry (IHC) experiments (Fig. 1H). Furthermore, the clinicopathological features of patients with PCa were investigated by analyzing the RNA-sequencing data from TCGA database. As Table 1 showed, high GILT expression was significantly correlated with higher T stage (*p* = 0.006), N stage (*p* < 0.001), PSA level (*p* = 0.004), and higher Gleason score (*p* < 0.001). Moreover, there was a poor progression-free interval in the high GILT expression group (Fig. 1I; Table 1).

**Table 1:** Correlations between clinicopathological parameters and GILT mRNA expression of PCa patients.

Characteristics	GILT mRNA expression		*p*-value
	Low (*n* = 250)	High (*n* = 261)	
Age, median (IQR)	60 (55, 65)	63 (58, 67)	**<0.001**^a^
T stage, *n* (%)			**0.006**^b^
T2	112 (22.7%)	77 (15.6%)	
T3	132 (26.7%)	162 (32.8%)	
T4	4 (0.8%)	7 (1.4%)	
N stage, *n* (%)			**<0.001**^b^
N0	180 (42.1%)	168 (39.3%)	
N1	24 (5.6%)	56 (13.1%)	
M stage, *n* (%)			0.262^c^
M0	225 (48.9%)	232 (50.4%)	
M1	0 (0%)	3 (0.7%)	
PSA (ng/ml), *n* (%)			**0.004**^b^
<4	212 (47.7%)	205 (46.2%)	
≥4	6 (1.4%)	21 (4.7%)	
Gleason score, *n* (%)			**<0.001**^c^
6	28 (5.6%)	18 (3.6%)	
7	149 (29.7%)	99 (19.8%)	
8	28 (5.6%)	37 (7.4%)	
9	44 (8.8%)	94 (18.8%)	
10	1 (0.2%)	3 (0.6%)	
PFI event, *n* (%)			**0.003**^b^
No	216 (43.1%)	191 (38.1%)	
Yes	34 (6.8%)	60 (12%)	
OS event, *n* (%)			0.754^c^
Alive	246 (49.1%)	245 (48.9%)	
Dead	4 (0.8%)	6 (1.2%)	

The GILT low and high-expression groups were divided by the median GILT expression value. Stattistically significant *p* values are shown in bold.

*IQR* interquartile range, *PSA* prostate-specific antigen, *PFI* progression-free interval, *OS* overall survival.

*p*^a^ Wilcoxon test.

*p*^b^ chi-squared test.

*p*^c^ Yates’ correction.

### GILT knockdown inhibits the invasion and migration of PCa cells in vitro and in vivo

**Fig. 2:**
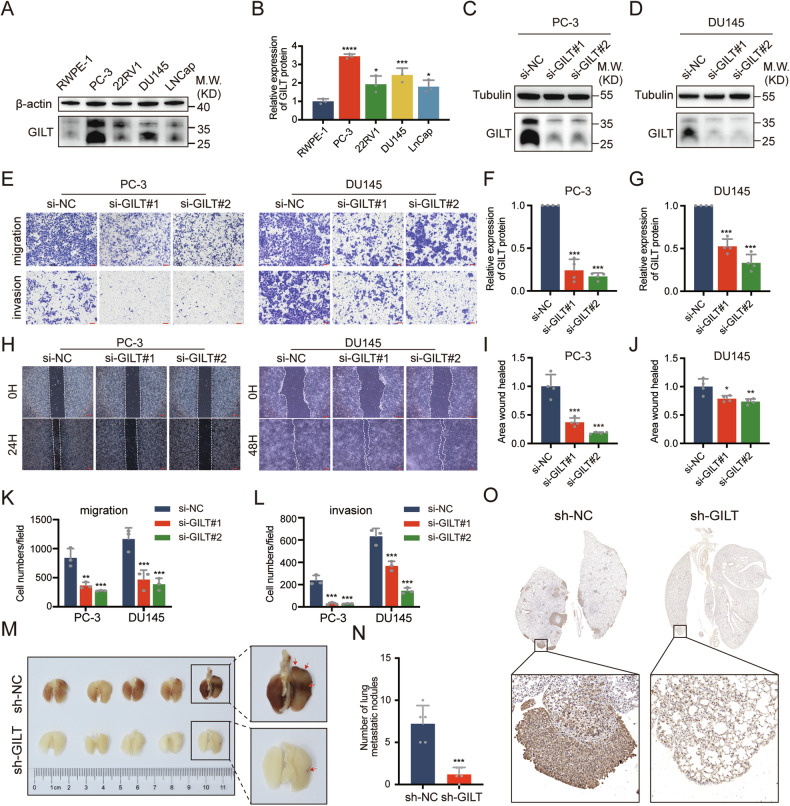
Knockdown of GILT inhibits the invasion and migration of PCa. **A**, **B** Western blot results indicate that the expression of GILT in various PCa cell lines is higher than that in normal prostate epithelial cells. The data are expressed as mean ± SD. *p < 0.05, ***p < 0.001, Student’s *t*-test, *n* = 3. **C**, **D** The knockdown efficiency is confirmed by Western blot. **E** Transwell assay results demonstrate significant inhibition of invasion and migration of PCa cells after GILT knockdown (scale bar, 100 μm). **F**, **G** The Western blot results in Fig. 2C, D are quantified. The data are expressed as mean ± SD. ***p < 0.001, Student’s *t*-test, *n* = 3. **H** Scratch assay shows significant inhibition of PCa cell invasion after GILT knockdown (scale bar, 200 μm). **I**, **J** Quantification of wound healing rate. The data are expressed as mean ± SD. *p < 0.05, **p < 0.01, ***p < 0.001, Student’s *t*-test, *n* = 3. **K**, **L** Quantitative analyses of migration and invasion assays. The data are expressed as mean ± SD. **p < 0.01, ***p < 0.001, Student’s *t*-test, *n* = 3. **M**, **N** Images of mouse lungs, and pulmonary metastatic nodules are recorded and quantified (red arrows indicate metastatic nodules). The data are expressed as mean ± SD. ***p < 0.001, Student’s *t*-test, *n* = 5. **O** Representative HE staining of mouse lung tissues. PCa prostate cancer, GILT gamma-interferon-inducible lysosomal thiol reductase, si-NC negative control of knockdown, sh short hairpin RNA, HE hematoxylin-eosin.

GILT exhibited significant overexpression in various PCa cell lines compared with normal prostate epithelial cells (RWPE-1), especially in PC-3 and DU145 cells (Fig. 2A, B). Additionally, in the prostate cancer cell lines, both the precursor (35 kDa) and mature form (25 kDa) of GILT were more distinctly visible than in tissue samples, and both forms were included in the semi-quantitative analysis. Consequently, two small interfering RNAs (siRNAs) targeting GILT were synthesized to explore its biological function in PCa cells, specifically in PC-3 and DU145 cells. Their efficacy was confirmed by RT-qPCR (Supplementary Fig. S2A) and Western blot analysis (Fig. 2C, D, F, G). GILT knockdown did not significantly affect PCa cell proliferation (Supplementary Fig. S1A–D). Scratch and transwell assays revealed significant inhibition in PCa cell invasion and migration following GILT knockdown (Fig. 2E, H, I–L). Next, a mouse model of lung metastasis was employed to assess the biological function of GILT in vivo. Using a GILT-shRNA plasmid containing the most efficient knockdown sequence, a PC-3 cell line with stable GILT knockdown was established and subsequently injected into mice via the tail vein. After 50 days, the mouse lungs were harvested for imaging (Fig. 2M), and metastatic nodules on the lung surface were quantified (Fig. 2N). The results indicated a significant reduction in lung metastasis due to GILT depletion. IHC confirmed metastatic nodules in the mouse lungs (Fig. 2O).

### GILT binds to and stabilizes cofilin

**Fig. 3:**
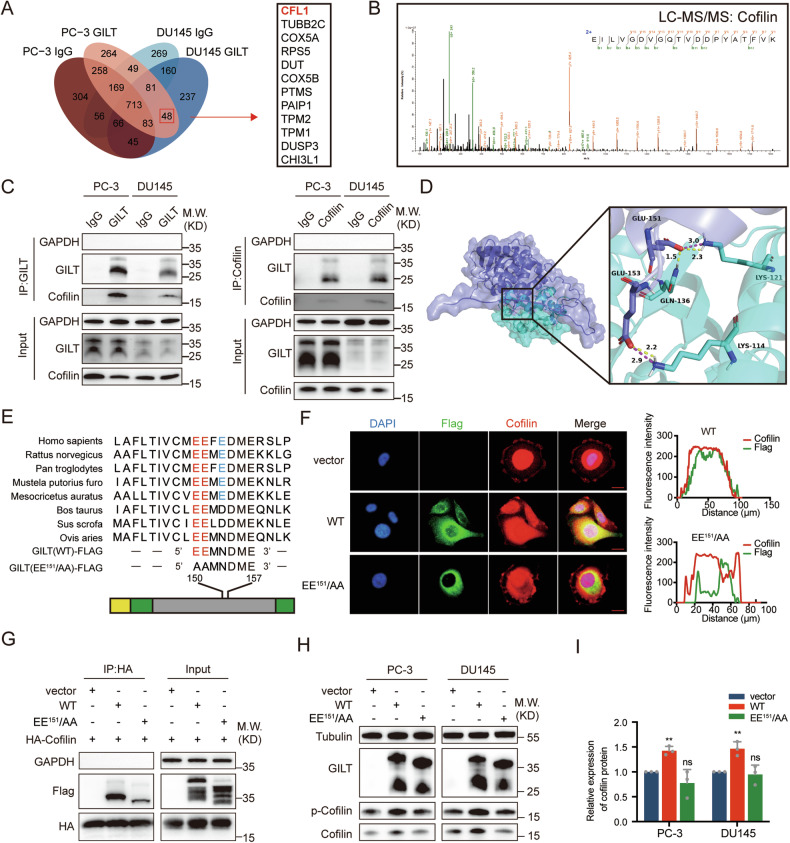
GILT directly interacts with cofilin and exerts a stabilizing effect. **A** The schematic of GILT-binding proteins in PCa cells, with CFL1 ranked first in abundance. **B** Detection of cofilin protein peptide in precipitates through liquid chromatography-tandem mass spectrometry (LC-MS/MS). **C** Immunoprecipitation in PCa cells using GILT antibody (left) and cofilin antibody (right), Western blot results confirm the physical interaction between GILT and cofilin in PCa cells. **D** Simulation graph of potential binding sites between GILT and cofilin. **E** Conservation of GILT sequences among different species (above), schematic illustration of different GILT constructs (below), with the yellow segment indicating the signal peptide and the green segment indicating the propeptide. **F** Immunofluorescence results demonstrate co-localization of wild-type GILT and cofilin in the cytoplasm of PC-3 cells (scale bar, 20 μm). However, this co-localization is abolished by the mutation of key amino acids (left). The fluorescence intensity was quantitatively analyzed along the longest axis of the cell (right). **G** Transfection of HA-tagged cofilin and different Flag-tagged GILT constructs into 293T cells. The coIP results confirm that mutation of key amino acids decreased the interaction between GILT and cofilin. **H**, **I** The Western blot results demonstrate that the expression of wild-type GILT in PCa cells increases cofilin protein levels, whereas the mutant fails to do so. The data are expressed as mean ± SD. **p < 0.01, Student’s *t*-test, *n* = 3. PCa prostate cancer, GILT gamma-interferon-inducible lysosomal thiol reductase, CFL1 cofilin, WT wild-type GILT, EE^151^/AA GILT mutant, LC-MS/MS liquid chromatography-tandem mass spectrometry, si-NC negative control of knockdown, ns no significance, Glu glutamate, Lys lysine, Gln glutamine, coIP Co-immunoprecipitation.

Further investigation was conducted into the biological mechanism underlying GILT’s promotion of PCa metastasis. Plasmids encoding both wild-type (WT) GILT and a GILT mutant (active site cysteine mutated to serine, SXXS) were constructed and transfected into PC-3 and DU145 cells (Supplementary Fig. S2B). Experimental results revealed that the capacity of GILT to reduce disulfide bonds did not influence its role in promoting PCa cell migration (Supplementary Fig. S2C, S2D). In addition, immunoprecipitation and mass spectrometry were used to identify proteins associated with GILT (Fig. 3A). A total of 48 GILT-binding proteins were identified, notably with cofilin as the primary interactor (Fig. 3B). Cofilin, a vital regulator of actin filament dynamics, is known for promoting actin filament depolymerization and accelerating turnover. The direct interaction between GILT and cofilin in PC-3 and DU145 cells was verified via Co-immunoprecipitation (coIP) and Western blot analysis (Fig. 3C).

Gramm docking simulations revealed that glutamate at positions 151 (Glu-151) and 153 (Glu-153) of GILT formed hydrogen bonds with cofilin, facilitating physical interaction; a three-dimensional docking model was subsequently generated (Fig. 3D). Protein sequence conservation analysis revealed a high degree of conservation for Glu-150 and Glu-151 in GILT across various species. Subsequently, the two glutamate residues were mutated to alanine to disrupt hydrogen bond formation (denoted as EE^151^/AA, hereafter for conciseness), and explore whether these were key interaction sites between GILT and cofilin (Fig. 3E). Plasmids containing Flag-tagged WT GILT or EE^151^/AA were transfected into PCa cells. Immunofluorescence confirmed the subcellular co-localization of WT GILT and cofilin, while Glu mutations attenuated this co-localization (Fig. 3F). Hemagglutinin (HA) antibodies were used for subsequent exogenous immunoprecipitation in 293T cells. Western blot outcomes showed that multiple bands were detected in the input fraction by the Flag antibody due to signal peptide cleavage, pre-peptide processing, and glycosylation modification [25–27]. The Flag antibody detected WT GILT signal in the immunoprecipitation complex pulled down with the HA antibody, whereas mutation of critical amino acid glutamate markedly reduced this physical interaction (Fig. 3G). Consistent with the immunoprecipitation results, WT GILT significantly increased cofilin protein levels in PCa cells, while mutation of critical amino acids eliminated this effect (Fig. 3H, I).

### GILT promotes PCa cell metastasis by stabilizing cofilin to facilitate actin severing and depolymerization

**Fig. 4:**
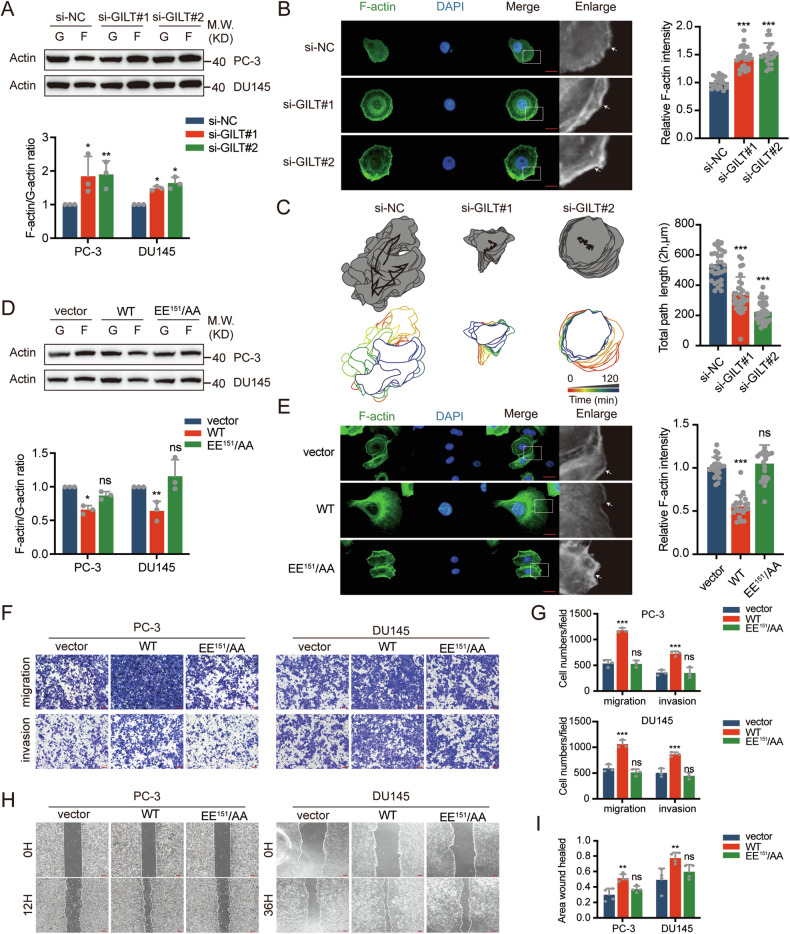
GILT promotes PCa cell metastasis by stabilizing cofilin. **A** Western blot results confirm that the level of F-actin increases while the level of G-actin decreases after GILT silencing in PCa cells, resulting in an increased F/G-actin ratio. The data are expressed as mean ± SD. *p < 0.05, **p < 0.01, Student’s *t*-test, *n* = 3. **B** GILT knockdown inhibits depolymerization of F-actin at the cell leading edge (white arrows) in PC-3 cells (scale bar, 20 μm). Statistical analysis revealed that the fluorescence intensity at the cell leading edge (5 μm) was higher than the overall cell fluorescence intensity, The data are expressed as mean ± SD. ***p < 0.001, Student’s *t*-test, *n* = 20. **C** Time-lapse microscopy was used to monitor cell movement. Based on the timeline, the morphology of cells at each time point was outlined, and the center points were connected into a continuous line. The results indicate that knocking down GILT inhibits the total path length of cell migration. The data are expressed as mean ± SD. ***p < 0.001, Student’s *t*-test, *n* = 30. **D** Overexpression of GILT in PCa promotes the depolymerization of F-actin, leading to a decrease in the F/G-actin ratio. Mutation of key amino acid residues abolished this effect. The data are expressed as mean ± SD. *p < 0.05, **p < 0.01, Student’s *t*-test, *n* = 3. **E** Immunofluorescence results demonstrated that overexpression of GILT in PC-3 cells promotes the depolymerization of F-actin at the leading edge (white arrows). Mutation of key amino acid residues abolished this promoting effect, with the fluorescence intensity at the cell edge (5 μm) lower than the overall cell fluorescence intensity (scale bar, 20 μm). The data are expressed as mean ± SD. ***p < 0.001, Student’s *t*-test, *n* = 20. **F**–**I** Results from transwell experiments (scale bar, 100 μm) and scratch assays (scale bar, 200 μm) indicate that overexpression of wild-type GILT enhances the invasion and migration abilities of PCa cells. However, this promoting effect is abolished when the binding capacity to cofilin is reduced. The data are expressed as mean ± SD. **p < 0.01, ***p < 0.001, Student’s *t*-test, *n* = 3. PCa prostate cancer, GILT gamma-interferon-inducible lysosomal thiol reductase, G G-actin, globular actin, F F-actin filamentous actin, WT wild-type GILT, EE^151^/AA GILT mutant, si-NC negative control of knockdown, ns no significance.

Studies have reported that the function of cofilin in cleaving actin is regulated by its phosphorylation and dephosphorylation at ser-3 [28]. Changes in the total protein levels of cofilin were observed after GILT knockdown or overexpression in PCa cells, while the levels of p-cofilin compared with total cofilin exhibited no significant differences between the two groups (Fig. 3H, Supplementary Fig. S4C, S4D). Cofilin controls G-actin and F-actin, promoting cell migration by reorganizing F-actin [29]. Knocking down GILT in PCa cells increases the ratio of F-actin to G-actin, shifting the actin balance towards polymerization (Fig. 4A). Phalloidin was then used to label F-actin, and immunofluorescence results showed that GILT knockdown in PC-3 cells caused F-actin to change from a relatively uniform fluorescence signal to a weak cytoplasmic signal and a bright signal at the cell edge (Fig. 4B). By randomly selecting 20 fields, we compared the fluorescence intensity at the cell edge (5 μm) with the total fluorescence intensity, and the results showed a significant increase in subcortical F-actin signal after GILT knockdown. Next, we visualized cell movement under time-lapse microscopy, outlining the center of each cell at different time points with a continuous line (Fig. 4C). It was found that GILT knockdown significantly inhibited the total path length of cell migration. Consistently, GILT overexpression in PCa cells increased the invasive and migratory abilities and significantly increased the protein levels of cofilin (Supplementary Fig. S3A, S3B), whereas cofilin knockdown reversed the promoting effect of GILT (Supplementary Fig. S3C–3G). The fluorescence signal of F-actin in the cytoplasm of PC-3 cells overexpressing GILT was significantly higher than that at the cell edge protrusions (Fig. 4E). Western blot results also showed a shift in the balance of actin towards G-actin in PC-3 and DU145 cells, whereas no change was observed in the EE^151^/AA group (Fig. 4D). Additionally, transwell and scratch assays confirmed that GILT binding to cofilin played a critical role in promoting PCa metastasis (Fig. 4F–I). Taken together, these results suggest that GILT regulates actin dynamics in PCa cells by stabilizing cofilin, thereby promoting PCa metastasis.

### GILT stabilizes cofilin by inhibiting its ubiquitin-proteasomal degradation

**Fig. 5:**
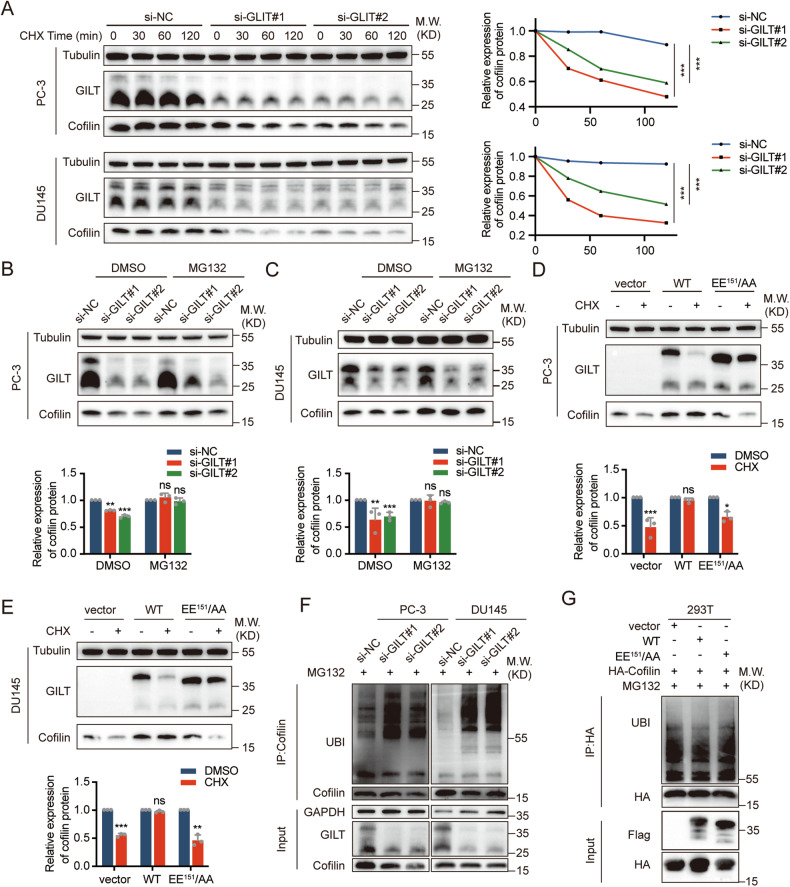
GILT stabilizes cofilin by inhibiting its ubiquitin-proteasomal degradation. **A** Treated PCa cells with CHX (10 μg/ml) after knocking down GILT, collected cells at specified time points, and subsequently performed Western blot to detect cofilin protein levels. The data are expressed as mean ± SD. ***p < 0.001, Student’s *t*-test, *n* = 3. **B**, **C** Cofilin protein levels were detected through western blot after treating PCa cells with MG132 (5 μM) following GILT knockdown, with DMSO as a control. The data are expressed as mean ± SD. **p < 0.01, ***p < 0.001, Student’s *t*-test, *n* = 3. **D**, **E** PCa cells transfected with different GILT constructs were treated with CHX for 4 h and cells were collected, followed by western blot to detect cofilin protein levels. The data are expressed as mean ± SD. *p < 0.05, **p < 0.01, ***p < 0.001, Student’s *t*-test, *n* = 3. **F** PCa cells with knockdown of GILT were treated with MG132 (5 μM), followed by immunoprecipitation with cofilin antibody, and western blot shows elevated levels of ubiquitination of cofilin. **G** Overexpressed HA-cofilin and different GILT constructs in 293T cells, followed by MG132 treatment and collection of the cells, and immunoprecipitation using the HA antibody showed a correlation between the ability of GILT to inhibit the ubiquitination of cofilin and its ability to bind to cofilin. GILT gamma-interferon-inducible lysosomal thiol reductase, PCa prostate cancer, WT wild-type GILT, EE^151^/AA GILT mutant, CHX cycloheximide, si-NC negative control of knockdown, ns no significance, min minutes.

Cofilin protein levels were strongly reduced, whereas the mRNA level of cofilin remained unaltered after GILT knockdown or overexpression in PCa cells (Supplementary Fig. S4A–S4D), suggesting that GILT might affect cofilin protein at the post-translational level. To understand how GILT affects cofilin protein stability, CHX was used to inhibit protein synthesis and measure changes in cofilin levels over time. Results showed that cofilin levels in GILT-knockdown cells decreased by half after 2 h of CHX treatment, significantly lower than in the control group (Fig. 5A). The control group’s cofilin protein level decreased by half after 4 h of CHX treatment, while no significant change was observed in PCa cells with overexpressed WT GILT. Treatment with MG132, a proteasome inhibitor, rescued the decreased cofilin protein levels caused by GILT knockdown (Fig. 5B, C). However, the stabilizing effect of GILT on the cofilin protein was abolished following the mutation of critical amino acids in the GILT binding site (Fig. 5D, E). These results suggested that the stabilizing effect of GILT on cofilin may be related to ubiquitination. Next, the ubiquitination levels of cofilin during GILT expression regulation were investigated. Immunoprecipitation results showed that the ubiquitination level of cofilin increased dramatically in PCa cells after GILT knockdown (Fig. 5F, Supplementary Fig. S4G). Conversely, GILT overexpression inhibited the ubiquitination of cofilin. The inhibitory effect of GILT on cofilin ubiquitination was reduced after the mutation of critical amino acids for the binding of GILT and cofilin (Fig. 5G, Supplementary Fig. S4H). Consistently with GILT, cofilin protein was overexpressed in PCa samples (Supplementary Fig. S4I, S4J). These results suggest that GILT inhibits cofilin degradation through the ubiquitin-proteasome pathway, thereby enhancing the stability of the cofilin protein.

### GILT stabilizes cofilin by inhibiting SRC-mediated phosphorylation of cofilin

**Fig. 6:**
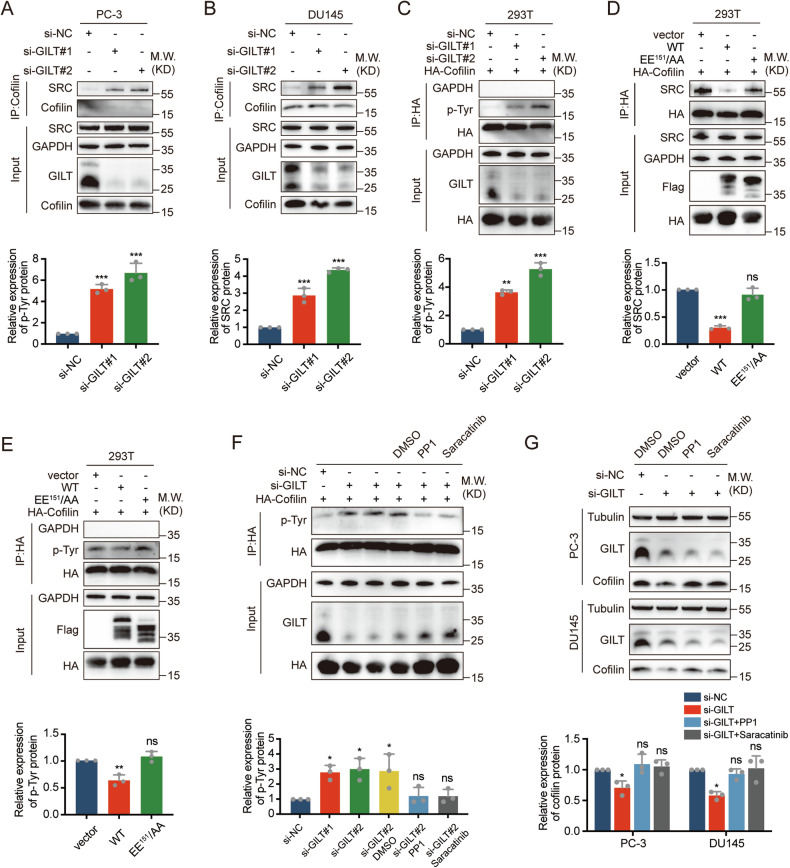
Combination of GILT inhibits ubiquitination degradation of cofilin induced by SRC phosphorylation. **A**, **B** Immunoprecipitation results confirm a significant increase in the binding of cofilin to SRC after knocking down GILT in PCa cells. The data are expressed as mean ± SD. ***p < 0.001, Student’s *t*-test, *n* = 3. **C** Expression of HA-cofilin in 293T cells was accompanied by GILT knockdown. Immunoprecipitation was then performed using HA antibody, and 4G10 antibody was used to detect the tyrosine phosphorylation level of cofilin. The data are expressed as mean ± SD. **p < 0.01, ***p < 0.001, Student’s *t*-test, *n* = 3. **D**, **E** Immunoprecipitation results showed that GILT could inhibit the binding of SRC to cofilin, reduce cofilin tyrosine phosphorylation levels, and mutation of key amino acids in the GILT binding site abolished this inhibitory effect. The data are expressed as mean ± SD. **p < 0.01, ***p < 0.001, Student’s *t*-test, *n* = 3. **F** GILT was knocked down after expression of HA-cofilin in 293T cells, which were then treated with SRC inhibitors PP1 (25 μM) and Saracatinib (10 μM). Immunoprecipitation results showed that SRC inhibitors could restore the increased tyrosine phosphorylation levels of cofilin caused by GILT knockdown. The data are expressed as mean ± SD. *p < 0.05, Student’s *t*-test, *n* = 3. **G** Western blot results showed that treatment with SRC inhibitors PP1 (25 μM) and Saracatinib (10 μM) in PCa cells could restore the decreased cofilin protein levels caused by GILT knockdown. The data are expressed as mean ± SD. *p < 0.05, Student’s *t*-test, *n* = 3. GILT gamma-interferon-inducible lysosomal thiol reductase, PCa prostate cancer, SRC Src family tyrosine kinase, WT wild-type GILT, EE^151^/AA GILT mutant, si-NC negative control of knockdown, ns no significance.

Research has identified a fragment of cofilin (amino acids 133 to 146) among the substrates of the SRC family kinase [30]. Yoo et al. confirmed that SRC mediates tyrosine phosphorylation of cofilin at the cellular level, leading to cofilin degradation through the ubiquitin-proteasome pathway [21]. Therefore, we hypothesize that there is spatial competition between GILT and SRC for binding to cofilin in PCa cells, which exerts the stabilizing effect of GILT on cofilin. First, the cofilin-specific antibody was used for immunoprecipitation, and Western blot results showed that the binding of SRC to cofilin increased markedly after GILT silencing (Fig. 6A, B), while the protein expression levels of SRC in the cells remained unaffected (Supplementary Fig. S4E, S4F). Subsequently, a phospho-tyrosine antibody (4G10) was used, and Western blot results demonstrated that the tyrosine phosphorylation level of cofilin increased substantially after GILT knockdown compared with the control group (Fig. 6C). WT GILT overexpression resulted in a significant reduction in the binding of SRC to cofilin and a decrease in the tyrosine phosphorylation level of cofilin. However, this effect was lost after mutation of key amino acids in the binding site (Fig. 6D, E). To validate the role of SRC-catalyzed tyrosine phosphorylation in this process, changes in cofilin phosphorylation and protein levels were compared after treatment with SRC inhibitors PP1 and Saracatinib following GILT knockdown. The results showed that PP1 or Saracatinib rescued the increased tyrosine phosphorylation level of cofilin caused by GILT knockdown (Fig. 6F). Meanwhile, the protein level of cofilin, which was decreased due to GILT knockdown, was restored by administering PP1 or Saracatinib in PC-3 and DU145 cells (Fig. 6G). Collectively, these data suggest that GILT competes with SRC for binding to cofilin in PCa cells, thereby inhibiting the degradation of phosphorylated cofilin through the ubiquitin-proteasome pathway.

## Discussion

The incidence of PCa is increasing rapidly with population aging and advances in medical knowledge [31]. Currently, PCa is the most frequently diagnosed cancer in men [1]. Timely and effective treatment generally leads to a more optimistic prognosis for individuals with early-stage PCa. However, mPCa accounts for up to 54% of newly diagnosed PCa cases in China [2], which represents a critical stage that significantly impacts the prognosis of patients with PCa. After a median duration of 18–24 months of ADT, almost all patients with mPCa progress to CRPC, leading to a significant reduction in the life expectancy of PCa patients and presenting significant medical challenges [32]. Therefore, exploring the mechanisms underlying the progression and spread of PCa is imperative.

GILT was initially discovered and identified in interferon-gamma (IFN-γ) treated monocytes and is crucial for intracellular protein internalization [4], lysosomal reduction, and MHC class II antigen presentation [33]. Recent studies show GILT has non-immune functions and potential clinical value in various cancers [8, 34–36]. Huang et al. found that the combination of GILT and other lysosome-related genes with Gleason scores accurately predicted the prognosis of patients with PCa [12]. Bao et al. discovered a significant correlation between GILT mutation and the prognosis of ADT [13]. As research on GILT progresses, researchers have shown that after GILT is synthesized as a precursor in ribosomes, its terminal peptide segments facilitate its subsequent protein folding and modification into a mature form [25]. Furthermore, investigations have identified the presence of soluble mature GILT in the supernatant of cell cultures [26]. Our study confirmed that GILT is overexpressed in PCa tissues and cell lines, with high GILT expression positively correlated with advanced tumor stage and poorer progression-free survival. These findings indicate that GILT may possess potential clinical significance as a biomarker for the diagnosis and prognosis of PCa.

Subsequent in vivo and in vitro functional experiments further confirmed the promoting effect of GILT on the invasion and migration of PCa cells. With the advancement in molecular biology techniques, the structural characteristics of GILT such as its signal peptides, pro-peptides, and glycosylation modifications, along with their corresponding functions, have gradually been revealed [25, 27]. GILT is characterized by an active-site CXXC motif and an enzymatic mechanism in which the active-site cysteine pair cooperates to reduce substrate disulfide bonds. First, we constructed plasmids encoding WT GILT or an active site mutation (SXXS) and transfected them into PC-3 and DU145 cells. Transwell assay and scratch healing experiments confirmed that the reducing ability of GILT did not affect its promotion of PCa cell migration. Subsequently, using immunoprecipitation and mass spectrometry, we confirmed an interaction between GILT and cofilin. This finding offers a new perspective on the role and mechanisms of GILT in tumor progression. As a major regulatory protein of actin with depolymerization and severing activity, cofilin plays a critical role in multiple cellular processes that require reorganization or rapid turnover of actin filaments [37]. Several studies have confirmed that cofilin promotes the invasion and migration of cancer cells in breast cancer, lung cancer, colorectal cancer, and pancreatic cancer [38–40]. Cofilin is subject to regulation in cells by various mechanisms, the primary one being phosphorylation at Ser-3 [14]. Our data confirmed that altering GILT expression affected the total protein level of cofilin in PCa cells without altering its Ser-3 phosphorylation or mRNA levels, indicating a novel post-translational modification by GILT. The target actin of cofilin exists in two forms: G-actin and polymerized F-actin, the latter is composed of aggregated G-actin. When cofilin is absent or its activity is inhibited, actin remains polymerized at the cell periphery and is unable to join the cellular actin pool and participate in the dynamic reorganization of F-actin that mediates the cell migration process [41]. By separating G-actin from F-actin, we found that the ratio of F-actin to G-actin changed significantly in PCa after regulating GILT expression. Meanwhile, immunofluorescence results revealed that GILT overexpression promoted the dynamic renewal of F-actin in lamellipodial protrusions at the cell edge, further affecting the formation of protrusions during the cell migration process. Combined with rescue experiments, we inferred that GILT stabilizes cofilin to regulate actin dynamics, promoting PCa metastasis. As a downstream-regulated protein of IFN-γ, our findings provide new insights into the malignant progression of PCa under low-grade inflammation conditions [42]. However, further research is needed to explore the outcomes of tumor cell responses and their immune microenvironment in the context of IFN-γ stimulation.

Subsequently, we further investigated the mechanism underlying the stabilizing effect of GILT on cofilin protein. In cells lacking the stabilizing effect of GILT, cofilin levels decreased significantly after CHX treatment. This reduction was reversed by the proteasome inhibitor MG132. Furthermore, immunoprecipitation clearly showed increased ubiquitination of cofilin in prostate cancer cells following GILT knockdown. Protein phosphorylation and ubiquitination are crucial post-translational regulatory mechanisms for proteins [43]. Recent evidence suggests that these two protein modifications may interact positively or negatively. Protein phosphorylation is a marker for proteins targeted to the ubiquitin-proteasome pathway [44]. A proteomic study identified a protein fragment of cofilin (amino acids 133 to 146) from substrates of the SRC kinase [30]. Yoo et al. confirmed that SRC mediates the phosphorylation of tyrosine 68 in cofilin, leading to cofilin degradation through the ubiquitin-proteasome pathway [21]. Therefore, we hypothesized that there is spatial competition between GILT and SRC for binding to cofilin in PCa cells, which exerts the stabilizing effect of GILT on cofilin. A cofilin-specific antibody isolated cofilin-related proteins, revealing increased SRC binding to cofilin and higher tyrosine phosphorylation after GILT knockdown. We further utilized SRC inhibitors PP1 and Saracatinib to confirm that blocking SRC kinase activity reverses the GILT knockdown effect. This suggests that GILT and SRC compete for cofilin binding, preventing the degradation of phosphorylated cofilin via the ubiquitin-proteasome pathway. Src family kinases are now recognized as key regulators of a complex signaling network that drives the development of castration resistance and bone metastases in PCa [45]. Numerous small molecule inhibitors targeting Src have been developed, and many have demonstrated encouraging results in preclinical studies [46]. However, despite some being tested in patients, these inhibitors have yet to deliver satisfactory outcomes for those with metastatic castration-resistant prostate cancer [47]. Our findings will provide a new therapeutic target for further investigation.

**Fig. 7:**
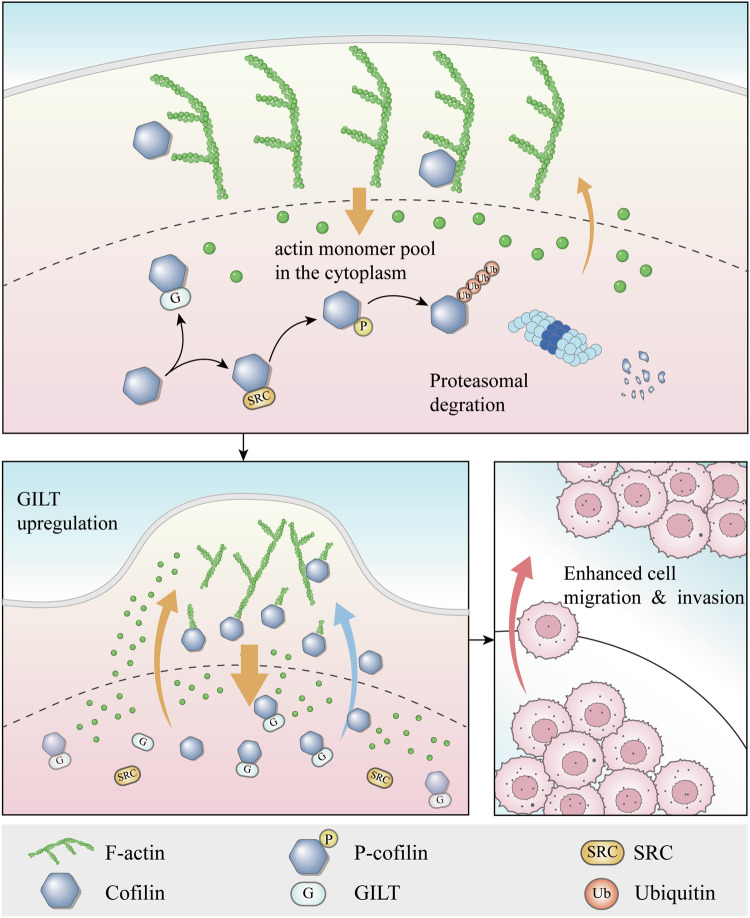
Schematic illustrating that GILT promotes PCa metastasis. GILT competitively binds to cofilin with SRC, preventing cofilin from entering the ubiquitin-proteasome pathway for degradation mediated by SRC-induced tyrosine phosphorylation, thereby stabilizing cofilin and further affecting G-actin/F-actin to promote PCa cell metastasis. PCa prostate cancer, GILT gamma-interferon-inducible lysosomal thiol reductase, G G-actin, globular actin, F F-actin, filamentous actin, SRC Src family tyrosine kinase, Ub ubiquitin.

In summary, the current study not only confirmed the high expression of GILT in PCa cells but also demonstrated its role in promoting the invasion and migration of PCa cells. Mechanistically, it was revealed that GILT competitively binds to cofilin with SRC, thus preventing cofilin from undergoing ubiquitin-proteasome pathway degradation mediated by SRC-induced tyrosine phosphorylation. This stabilization of cofilin further influences the dynamic balance and renewal of G-actin/F-actin, playing a key role in promoting cancer cell migration (Fig. 7). Our research provides a new perspective on the role of GILT in promoting PCa metastasis and may be a novel target for PCa diagnosis and clinical treatment.

## Materials and methods

### Patients and specimens

The paired PCa and adjacent non-cancerous prostate (ANP) tissue specimens were collected from patients undergoing radical prostatectomy at Jinshan Hospital, Fudan University. Pathological diagnoses were independently validated by two pathologists. All tissues were flash-frozen in liquid nitrogen within 15 min after surgical excision and stored at –80 °C. This study was approved by the Ethics Committee of Jinshan Hospital, Fudan University. Written consent was obtained from each patient, and all methods were performed in accordance with relevant guidelines and regulations.

### Cell lines

Cell lines, including HEK293T, human PCa lines (DU145, PC-3, 22RV1, LNCaP), and normal human prostate epithelial cells (RWPE-1), were sourced from the FuHeng Cell Center (FuHeng, Shanghai, China). HEK293T and DU145 were cultured in DMEM (Gibco, Thermo Fisher Scientific, MA, USA), PC-3 in DMEM/F12 (HyClone, GE Healthcare Life Science, UT, USA), and LNCaP and 22RV1 in RPMI-1640 (Gibco, Thermo Fisher Scientific, USA). RWPE-1 cells were cultured in keratinocyte SFM (Gibco, Thermo Fisher Scientific, USA). All media were supplemented with 10% fetal bovine serum (FBS; BI, Beit Haemek, Israel), and cells were maintained at 37 °C with 5% CO_2_. For some experiments, cells were incubated with MG132 (5 μM, MCE, NJ, USA), PP1 (25 μM, Beyotime, Shanghai, China), Saracatinib (10 μM, Beyotime, Shanghai, China), and cycloheximide (CHX, 10 μg/ml, MCE, NJ, USA) for 2–5 h before lysis.

### RNA interference

Appropriate numbers of PC-3 and DU145 cells were seeded separately in 6-well plates. Transfection was performed 24 h later when cells reached 60% confluence. Using the X-tremeGENE siRNA transfection reagent (Roche, Mannheim, Germany), transfection of si-GILT, si-cofilin, and negative control siRNA (GenePharma, Shanghai, China) was conducted in accordance with the manufacturer’s protocol. The sequences of the siRNAs are listed in Supplementary Table S1.

### Plasmid construction and transfection

The most efficient si-GILT and si-NC were synthesized and cloned into short hairpin RNA (shRNA) using the PLKO.1 vector (Genewiz, Suzhou, China). The sequences of sh-GILT and sh-NC are detailed in Supplementary Table S1. Using the PlvxIRES-Puro vector (Genewiz, Suzhou, China), full-length GILT cDNA was synthesized and cloned. A mock vector was employed as the control. HEK293T cells underwent transfection with Plvx-IRES-Puro or PLKO.1, pSPAX2, and pMD2.G plasmids, using lipoD293 transfection reagent (SignaGene Laboratories, Rockville, USA), in accordance with the manufacturer’s protocol. Post 24, 48, and 72 h of transfection, the lentivirus-containing supernatant was harvested via centrifugation at 1000 × *g* for 5 min. PC-3 and DU145 cells, seeded separately in 6-well plates, were cultured to 30–50% confluence prior to transfection. The lentiviral supernatant was mixed with complete culture medium in a 1:1 ratio. Subsequently, cells were treated with 10 μg/mL polybrene (Sigma-Aldrich, St. Louis, USA). Following 48 h of incubation, 3 μg/mL puromycin was introduced to the culture medium for the selection of puromycin-resistant cells.

### RNA extraction and reverse transcription quantitative PCR (RT-qPCR)

Total RNA from tissues or cells was extracted using the RNA-Quick purification kit (Yishan, Shanghai, China). Quantification and quality evaluation of the RNA samples were conducted using the NanoDrop system (Thermo Fisher Scientific, USA). For reverse transcription, 500 ng of RNA was used in a 10 μL reaction system with PrimeScript RT Master Mix (Takara, Shiga, Japan). The resultant complementary DNA (cDNA) was diluted using diethylpyrocarbonate-treated water. Polymerase chain reaction (PCR) was conducted in three rounds on the ABI 7300 real-time PCR system (Thermo Fisher Scientific, USA), utilizing the BeyoFast SYBR Green qPCR Mix (Beyotime, Shanghai, China) as per the manufacturer’s instructions. Cycling parameters included an initial denaturation at 95 °C for 2 min, 40 cycles of 95 °C denaturation for 15 s, and annealing/extension at 60 °C for 30 s. The PCR results were analyzed through the ΔΔCT method, with primer sequences detailed in Supplementary Table S2.

### Western blot

Western blot analysis was performed as previously described [48]. All bands were quantified using ImageJ. To allow for a semi-quantitative analysis, the signal intensity of target proteins was normalized by the loading control (GAPDH, Tubulin, or β-actin), and the data were compared and normalized to the control group. Antibody information is listed in Table S3.

### Immunohistochemistry

Tissues were fixed in formalin overnight, embedded in paraffin blocks, and sectioned. GILT primary antibody was diluted 1:500. Slides were co-incubated with primary antibody overnight in a humidity chamber, followed by incubation with horseradish peroxidase (HRP)-conjugated secondary antibody for 2 h at room temperature. DAB was used to visualize the immune complex and the nuclei were counterstained with haematoxylin for contrast. Images were captured using Pannoramic DESK (3D Histech, Budapest, Hungary). Immunohistochemistry (IHC) results were evaluated by two independent pathologists according to the methodology described in previous studies [49]. Antibodies used are listed in Table S3.

### Immunostaining

Cells were seeded in a 96-well plate and cultured overnight. After 24 h, when the cells adhered stably to the surface, they were fixed with 4% paraformaldehyde (PFA) for 15 min, followed by permeabilization using phosphate-buffered saline (PBS) containing 0.1% Triton X-100 for 15 min. Subsequently, cells were blocked with PBS containing 5% bovine serum albumin and 0.1% Tween20 for 1 h at room temperature. After blocking, cells were incubated with primary antibodies (see Table S3) at 4 °C overnight. Subsequently, cells were incubated with Alexa Fluor 555-conjugated secondary antibody for 1 h at room temperature. F-actin was stained with iFluor 488-conjugated phalloidin (Yeasen, Shanghai, China). 4′,6-Diamidino-2-phenylindole (DAPI) was used for nuclear staining. All images were taken with an inverted fluorescence microscope (Olympus, Tokyo, Japan).

### Migration, invasion, and wound healing assays

In the migration experiments, DU145 (6 × 10^4^/well) and PC-3 (12 × 10^4^/well) cells were fully suspended in a serum-free culture medium and then added to the upper chamber of the Transwell plate (Corning, NY, USA). At the same time, complete culture medium containing 20% fetal bovine serum was added to the lower chamber of the Transwell plate. After the specified incubation periods-24 h for DU145, and 48 h for PC-3 cells (reduced by 12 h for functional experiments involving GILT overexpression), we fixed the cells that had migrated to the underside of the upper chamber with 4% paraformaldehyde for 30 min. The cells were then stained with crystal violet for an additional 30 min. Migration cells were counted in three random fields using an inverted microscope (Olympus, Tokyo, Japan). For the invasion experiments, the bottom surface of the Transwell upper chamber was pre-coated with diluted Matrigel (Corning, NY, USA), and the remaining steps were similar to the migration experiments.

The wound healing experiments were conducted as follows. Cells were seeded in a 6-well plate, and when they reached 90% confluency, a scratch was made using a 200 μL pipette tip. The initial scratch width was recorded, and the width of the scratch was measured after 24 h for PC-3 cells and 48 h for DU145 cells (reduced by 12 h for functional experiments involving GILT overexpression). The percentage of wound healing was calculated using the following formula: (initial scratch width - width of the scratch after healing)/initial scratch width × 100%.

### Co-immunoprecipitation assay

For the co-immunoprecipitation experiment, the co-immunoprecipitation (coIP) kit (BerSinBio, Guangzhou, China) was used according to the manufacturer’s protocol. Briefly, 2 × 10^7^ cells were harvested and lysed. Antibodies (5 mg) were mixed with the cell lysate and rotated at 4 °C overnight, followed by incubation with Protein A/G magnetic beads at room temperature for 3 h. Finally, the bound proteins were eluted from the beads and the protein samples were used for liquid chromatography-tandem mass spectrometry (LC-MS/MS) and Western blot. The antibodies utilized are listed in Table S3.

### In vivo study

The animal part of the study was approved by the Animal Research Committee of Fudan University. After appropriate processing, PC-3 cells (2 × 10^6^) were fully suspended in 200 µl PBS and injected into the tail vein of NOD/SCID mice. At 8 weeks after injection, the nude mice were sacrificed and perfused with formaldehyde. Subsequently, the lungs of the nude mice were excised and fixed with formaldehyde. After counting the number of metastatic nodules, the lungs were embedded in paraffin and sectioned. After dewaxing, the paraffin sections were stained with haematoxylin and eosin.

### F- and G-actin fractionation

The ratio of F-actin to G-actin was determined using Western blot, as previously described [50]. F-actin is insoluble, while G-actin is soluble. Cells were collected with a cell scraper and centrifuged at 1000 × *g* for 5 min at 4 °C after two washes with ice-cold PBS. Then the cell pellet was resuspended in cold lysis buffer 1 (10 mM K_2_HPO_4_, 100 mM NaF, 50 mM KCl, 2 mM MgCl_2_, 1 mM EGTA, 0.2 mM DTT, 0.5%Triton X-100, 1 mM sucrose, pH 7.0), homogenized and centrifuged at 15,000 × *g* for 30 min. The supernatant was collected and measured for the presence of G-actin.

The insoluble F-actin was resuspended in buffer 2 (1.5 mM guanidine hydrochloride, 1 mM sodium acetate, 1 mM CaCl_2_, 1 mM ATP, 20 mM Tris-HCl, pH 7.5) and incubated on ice for 1 h with gentle mixing every 15 min to convert it into soluble G-actin. The samples were then centrifuged at 15,000 × *g* for 30 min, and the F-actin in the supernatant was measured. The supernatant (G-actin) and pellet (F-actin) samples were taken in proportion and subjected to Western blot analysis using a specific actin antibody (see Table S3).

### Time-lapse imaging

An appropriate number of the control group and GILT-knockdown PC-3 cells were seeded in a 12-well plate. After 24 h, cells were imaged using a confocal microscope (Agilent, CA, USA) equipped with a motorized stage and a 37 °C cultivation system. The imaging data were collected from captured videos for 2–3 h (one frame every 3 min).

### Microarray analysis

Five pairs of PCa tissues and their ANP tissues were analyzed using microarray assays to identify dysregulated mRNAs. Sample labeling and array hybridization were performed according to the Agilent One-Color Microarray-Based Gene Expression Analysis protocol (Agilent, CA, USA). For the subsequent data analysis, we utilized the R package “limma”. Differentially expressed mRNAs were identified based on thresholds of fold change ≥1.5 and *p* values <0.05.

### Bioinformatics analysis

To explore the mechanisms underlying the progression of PCa, we integrated our microarray data with publicly available datasets. We utilized the TCGA_GTEx-PRAD dataset from UCSC XENA (https://xenabrowser.net/datapages/), which includes 152 samples of normal prostate tissue and 496 samples of PCa tissue. Additionally, we incorporated two datasets from the Gene Expression Omnibus (GEO, https://www.ncbi.nlm.nih.gov/geo/): one consisting of 10 primary PCa samples and 20 metastatic PCa samples (GSE6752) [23], and another comprising 8 untreated PCa samples and 40 CRPC samples (GSE101607) [24].

### Prediction and analysis of protein interaction

Regarding the prediction of the interaction between GILT and cofilin, the X-ray crystal structure of cofilin (5L6W) was obtained from the Protein Data Bank (https://www.rcsb.org/), and the predicted structure of GILT was obtained from AlphaFoldDB (https://alphafold.ebi.ac.uk/). To ensure the accuracy of the docking results, we subsequently performed manual optimization operations, such as desolvation and hydrogenation, on the two protein structures using AutoDockTools-1.5.7 [51]. Protein-protein docking was then conducted using the docking server (GRAMM) [52, 53]. Subsequently, the obtained protein-protein complexes underwent manual optimization operations, including desolvation and hydrogenation, using AutoDockTools-1.5.7. Finally, protein-protein interaction predictions were carried out using PyMOL (Schrödinger, NY, USA), and a protein-protein interaction diagram was generated.

### Statistical analysis

All experiments in this study were conducted at least three times. Statistical analysis was performed using GraphPad Prism 8 (GraphPad, San Diego, CA, USA). Data are presented as the mean ± standard deviation. Student’s *t*-test, Wilcoxon matched-pairs signed-rank test, one-way analysis of variance (one-way ANOVA), and Mann–Whitney U test were used according to actual conditions. Survival was analyzed by the Kaplan–Meier method. The levels of significance were set at: *p < 0.05, **p < 0.01, and ***p < 0.001.

## Supplementary information

Supplemental material

Original full length western blots

## Data Availability

All the data generated or analyzed in this study are included in this published article and its additional files.
