# Supplementary material for: GILT stabilizes cofilin to promote the metastasis of prostate cancer
Source: Cell Death Discov. 2025 Jan 16;11:10. doi: 10.1038/s41420-025-02288-0 (PMC11739388; doi:10.1038/s41420-025-02288-0)
Supplement: Supplementary file 1 — Supplemental material [file 41420_2025_2288_MOESM1_ESM.docx]

Supplementary Information

The Supplementary Material contains data on the impact of GILT on prostate cancer cell proliferation and the stabilization mechanism of GILT on the cofilin protein, as well as details regarding the siRNA, primers, and antibodies employed in the experimental procedures.
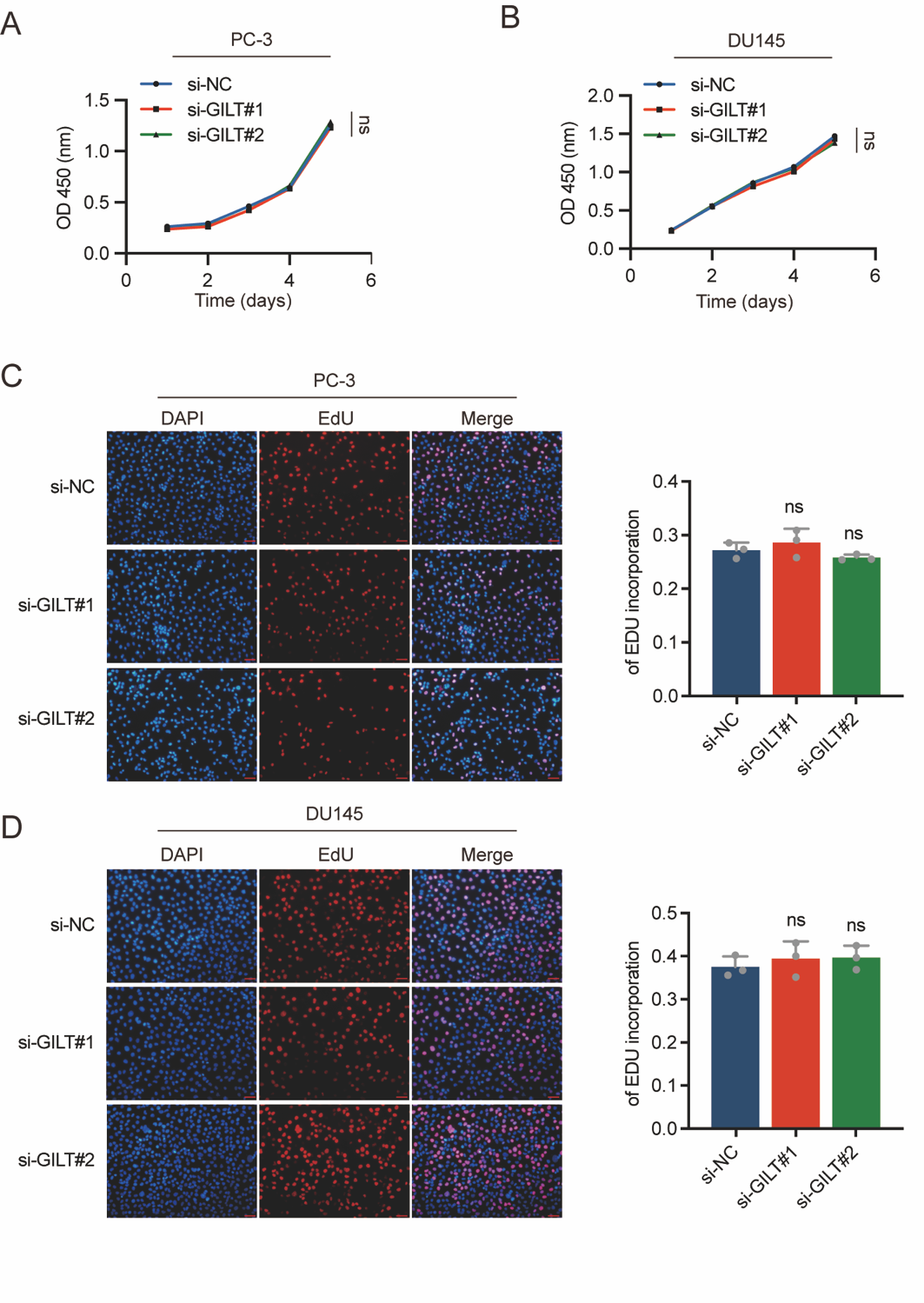


**Supplementary Fig S1**. Effects of GILT knockdown on PCa cell proliferation *in vitro*. (A, B) Cell proliferation rate of PC-3 and DU145 cells evaluated by CCK-8 assay upon GILT knockdown. The data are expressed as mean ± SD. Student’s t-test, n = 3. (C, D) The effects of GILT knockdown on PCa cell viability were detected by EdU assay (Scale bar, 100μm). The data are expressed as mean ± SD. Student’s t-test, n = 3. Abbreviations: PCa, prostate cancer; GILT, gamma-interferon-inducible lysosomal thiol reductase; si-NC, negative control of knockdown; ns, no significance; CCK-8, Cell Counting Kit-8.


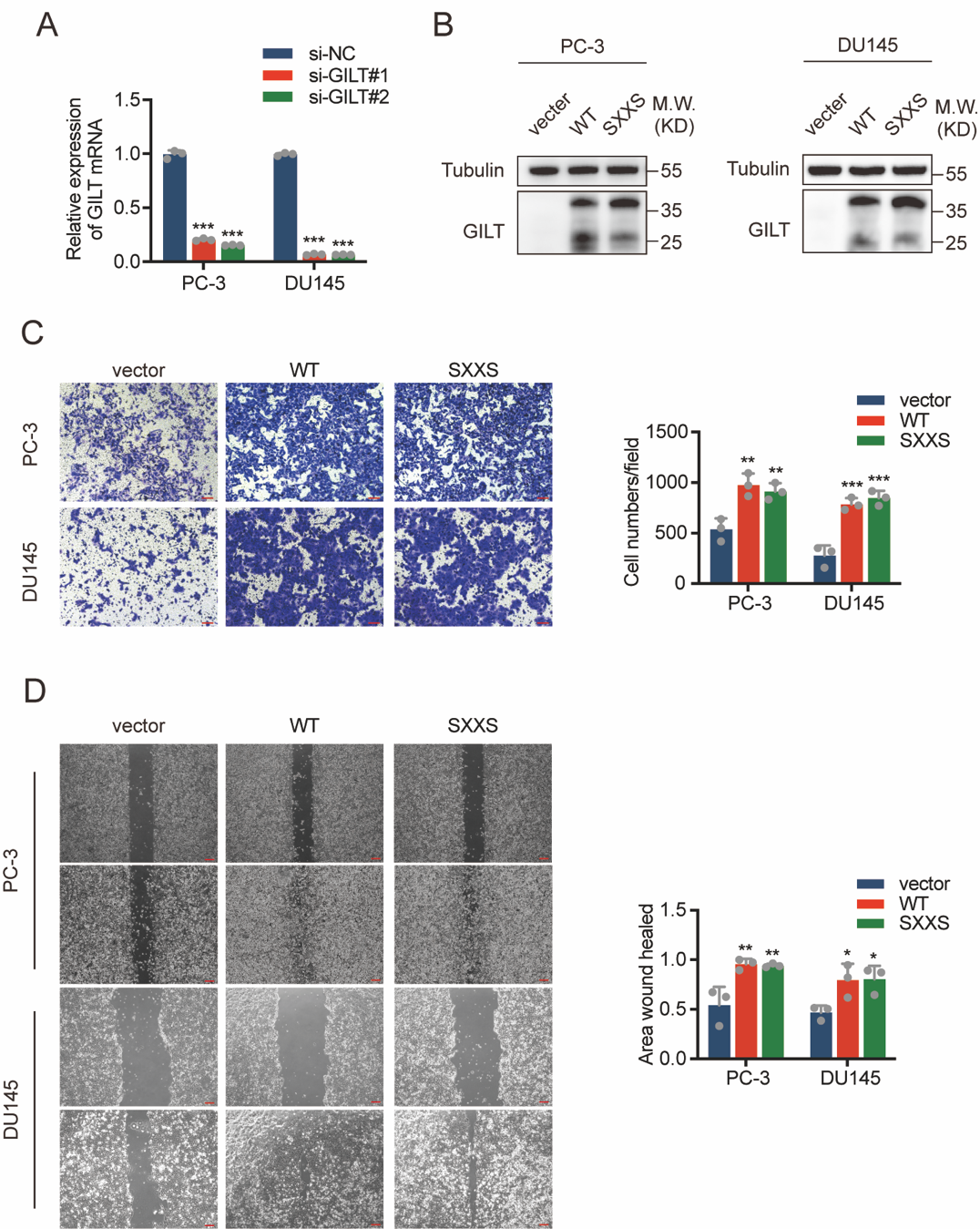


**Supplementary Fig S2**. GILT shows a promoting effect on PCa cell metastasis. (A) Knockdown efficiency of GILT was determined by RT-qPCR. The data are expressed as mean ± SD. *** *p* < 0.001, Student’s t-test, n = 3. (B) Western blot analysis was used to confirm the overexpression of GILT wild type or GILT mutant (SXXS). (C, D) PC-3 and DU145 cells migration (scale bar, 100μm) and scratch assay (scale bar, 200μm) following transfected GILT wild type or GILT mutant (SXXS). The data are expressed as mean ± SD. * *p* < 0.05, ** *p* < 0.01, *** *p* < 0.001, Student’s t-test, n = 3. Abbreviations: PCa, prostate cancer; GILT, gamma-interferon-inducible lysosomal thiol reductase; si-NC, negative control of knockdown; WT, wild-type GILT; SXXS, GILT mutant; RT-qPCR, reverse transcription quantitative PCR.


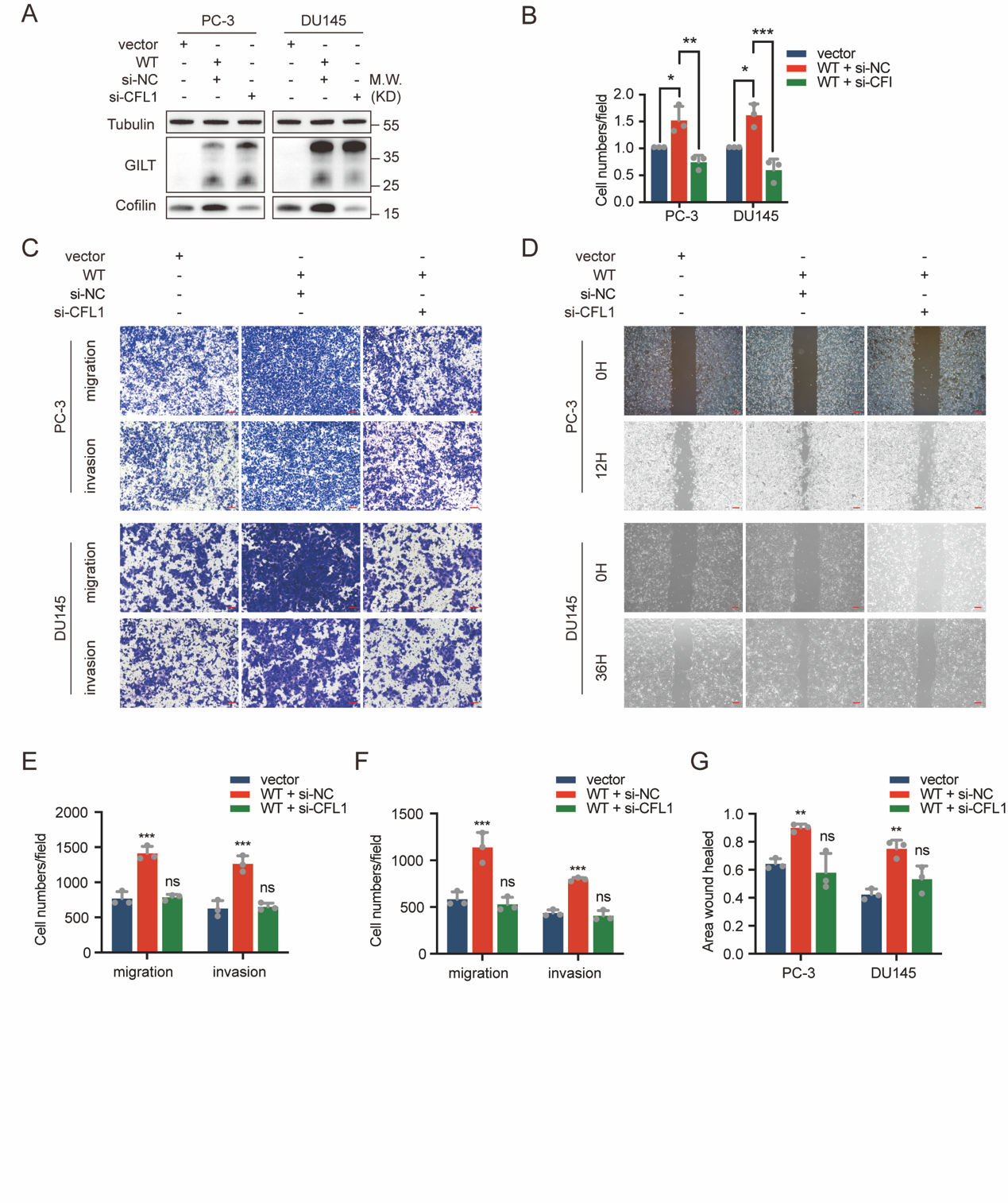


**Supplementary Fig S3**. GILT promotes PCa cell metastasis via cofilin. (A, B) The cofilin protein levels were determined after transfection of GILT and knockdown of cofilin. The data are expressed as mean ± SD. * *p* < 0.05, ** *p* < 0.01, *** *p* < 0.001, Student’s t-test, n = 3. (C, D) Rescue assays on cell migration and invasion were evaluated by transwell (scale bar, 100μm) and scratch assays (scale bar, 200μm). (E, F) Quantitative analysis of Fig S4C. The data are expressed as mean ± SD. *** *p* < 0.001, Student’s t-test, n = 3. (G) Quantitative analysis of wound-healing index. The data are expressed as mean ± SD. ** *p* < 0.01, Student’s t-test, n = 3. Abbreviations: PCa, prostate cancer; GILT, gamma-interferon-inducible lysosomal thiol reductase; CFL1, cofilin 1; WT, wild-type GILT; si-NC, negative control of knockdown; ns, no significance.


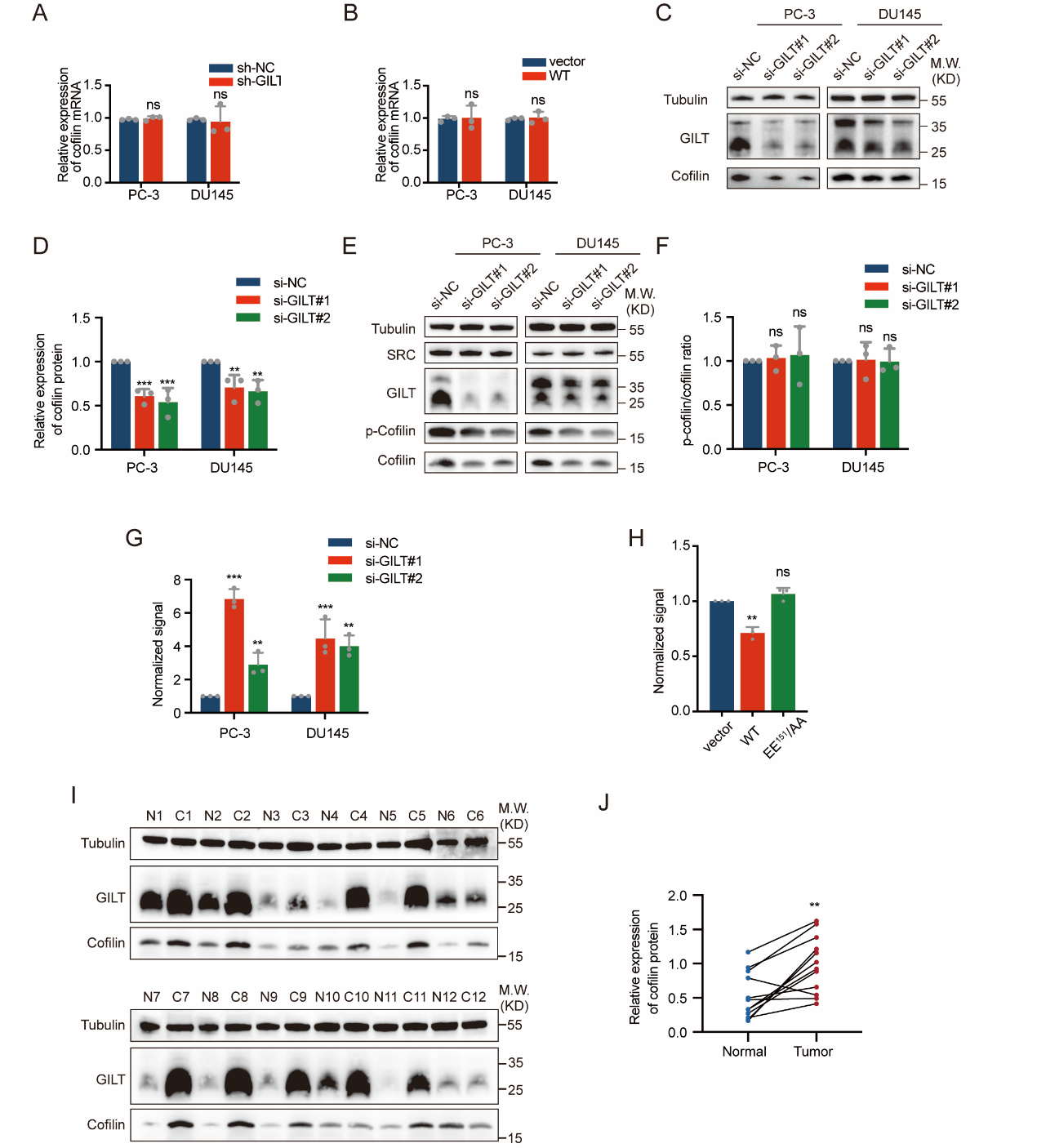


**Supplementary Fig S4**. GILT has a stabilizing effect on cofilin protein. (A, B) Cofilin mRNA levels were analyzed by RT-qPCR after GILT knockdown or overexpression. The data are expressed as mean ± SD. Student’s t-test, n = 3. (C, D) Cofilin protein levels were analyzed through western blot after GILT knockdown. The data are expressed as mean ± SD. ** *p* < 0.01, *** *p* < 0.001, Student’s t-test, n = 3. (E, F) The Ser-3 phosphorylation of cofilin after knockdown GILT was determined by Western analysis. The data are expressed as mean ± SD. Student’s t-test, n = 3. (G, H) The semi-quantitative analysis of cofilin ubiquitination after GILT knockdown or overexpression. (I, J) The cofilin protein level was measured in 12 pairs of PCa and corresponding adjacent normal prostate samples. The data are expressed as mean ± SD. ** *p* < 0.01, Student’s t-test, n = 12. Abbreviations: PCa, prostate cancer; GILT, gamma-interferon-inducible lysosomal thiol reductase; si-NC, negative control of knockdown; Ser, serine; SRC, Src family tyrosine kinase; ns, no significance; RT-qPCR, reverse transcription quantitative PCR.

**Supplementary Table S1.** siRNAs used in this study.

| **Gene** | **Sequences (5’-3’)** |
| --- | --- |
| GILT - 1 | GCGTTAGACTTCTTTGGGAAT |
| GILT - 2 | GCTTGTCAATGTGACCCTCTA |
| Cofilin | GCTTGTCAATGTGACCCTCTA |
| SRC | GCTCGGCTCATTGAAGACAAT |

Abbreviations: GILT, Gamma-interferon-inducible lysosomal thiol reductase; SRC, Src family tyrosine kinase.

**Supplementary Table S2.** Primers used in this study.

| **Primer** | **Direction** | **Sequences** |
| --- | --- | --- |
| GILT | Forward | 5'-GGCCTGCGTGTTGGATGAA-3' |
|  | Reverse | 5'-GGGCACATACTCGTGTGGT-3' |
| Cofilin | Forward | 5'-TTCAACGACATGAAGGTGCGT-3' |
|  | Reverse | 5'-TCCTCCAGGATGATGTTCTTCT-3' |
| SRC | Forward | 5'-GAGCGGCTCCAGATTGTCAA-3' |
|  | Reverse | 5'-CTGGGGATGTAGCCTGTCTGT-3' |
| β-actin | Forward | 5'-CATGTACGTTGCTATCCAGGC-3' |
|  | Reverse | 5'-CTCCTTAATGTCACGCACGAT-3' |
| GAPDH | Forward | 5'-GAACGGGAAGCTCACTGGCAT-3' |
|  | Reverse | 5'-GTCCACCACCCTGTTGCTGTAG-3' |

Abbreviations: GILT, Gamma-interferon-inducible lysosomal thiol reductase; SRC, Src family tyrosine kinase; GAPDH, glyceraldehyde-3-phosphate dehydrogenase.

**Supplementary Table S3.** Antibodies used in this study.

| **Antibody** | **Source** | **Cat No** | **Dilution used** |
| --- | --- | --- | --- |
| GILT | Proteintech | 11597-1-AP | 1:2000 |
| SRC | Abmart | T56605 | 1:2000 |
| Cofilin | Santa Cruz | sc-376476 | 1:500 |
| Phospho-Cofilin (Ser3) | Cell Signaling Technology | 3311 | 1:1000 |
| Phospho-Tyrosine (4G10) | Cell Signaling Technology | 96215 | 1:1000 |
| β-actin | Cell Signaling Technology | 8457 | 1:1000 |
| GAPDH | Cell Signaling Technology | 5174 | 1:1000 |
| HRP conjugated Goat Anti-Rabbit IgG | Cell Signaling Technology | 7074 | 1:1500 |
| HRP conjugated Goat Anti-Mouse IgG | Cell Signaling Technology | 7076 | 1:1500 |
| HRP conjugated Goat Anti-Rabbit IgG (IHC) | Servicebio | G1215-200T | 1:200 |
| Alexa Fluor 488 conjugated Goat anti-Rabbit IgG | ZSGB-BIO | ZF-0511 | 1:200 |
| Alexa Fluor 594 conjugated Goat anti-Mouse IgG | ZSGB-BIO | ZF-0513 | 1:200 |
| iFluorTM 488 phalloidin | YEASEN | 40736ES75 | 1:1000 |

Abbreviations: GILT, Gamma-interferon-inducible lysosomal thiol reductase; SRC, Src family tyrosine kinase; GAPDH, glyceraldehyde-3-phosphate dehydrogenase; IHC, immunohistochemistry; 4G10, phospho-tyrosine antibody; Ser, serine.
