# Supplementary material for: GILT stabilizes cofilin to promote the metastasis of prostate cancer
Source: Cell Death Discov. 2025 Jan 16;11:10. doi: 10.1038/s41420-025-02288-0 (PMC11739388; doi:10.1038/s41420-025-02288-0)

Fig 1G

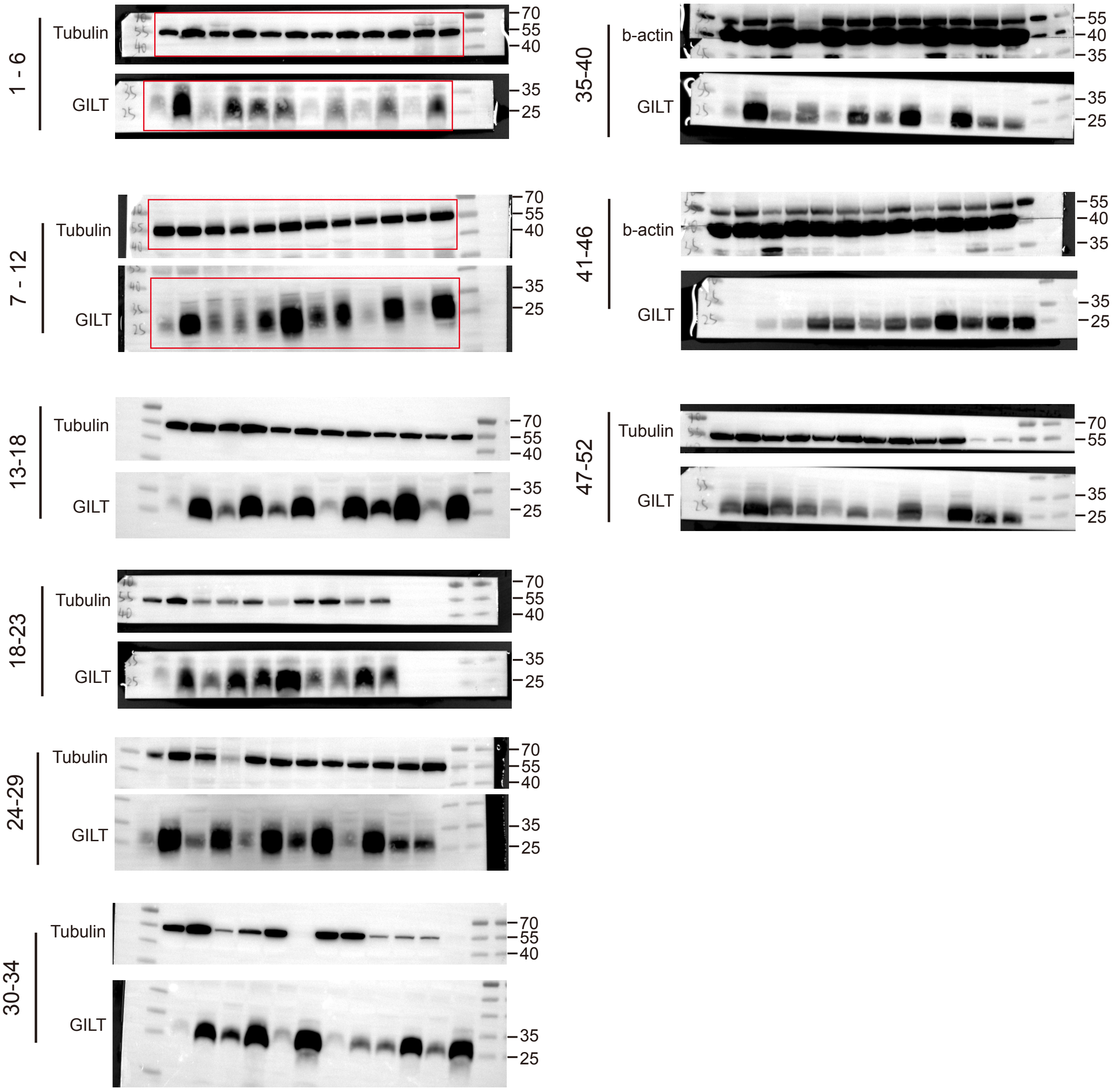
1 - 6
Tubulin
GILT
70
55
40
35
25
7 - 12
13-18
18-23
24-29
30-34
35-40
b-actin
41-46
47-52

Fig 2A

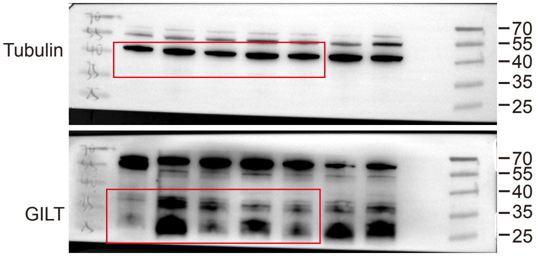

Fig 2C

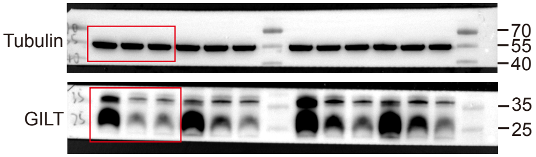

Fig 2D

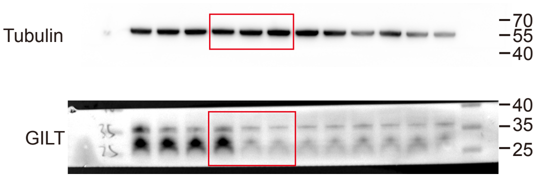

Fig 3C

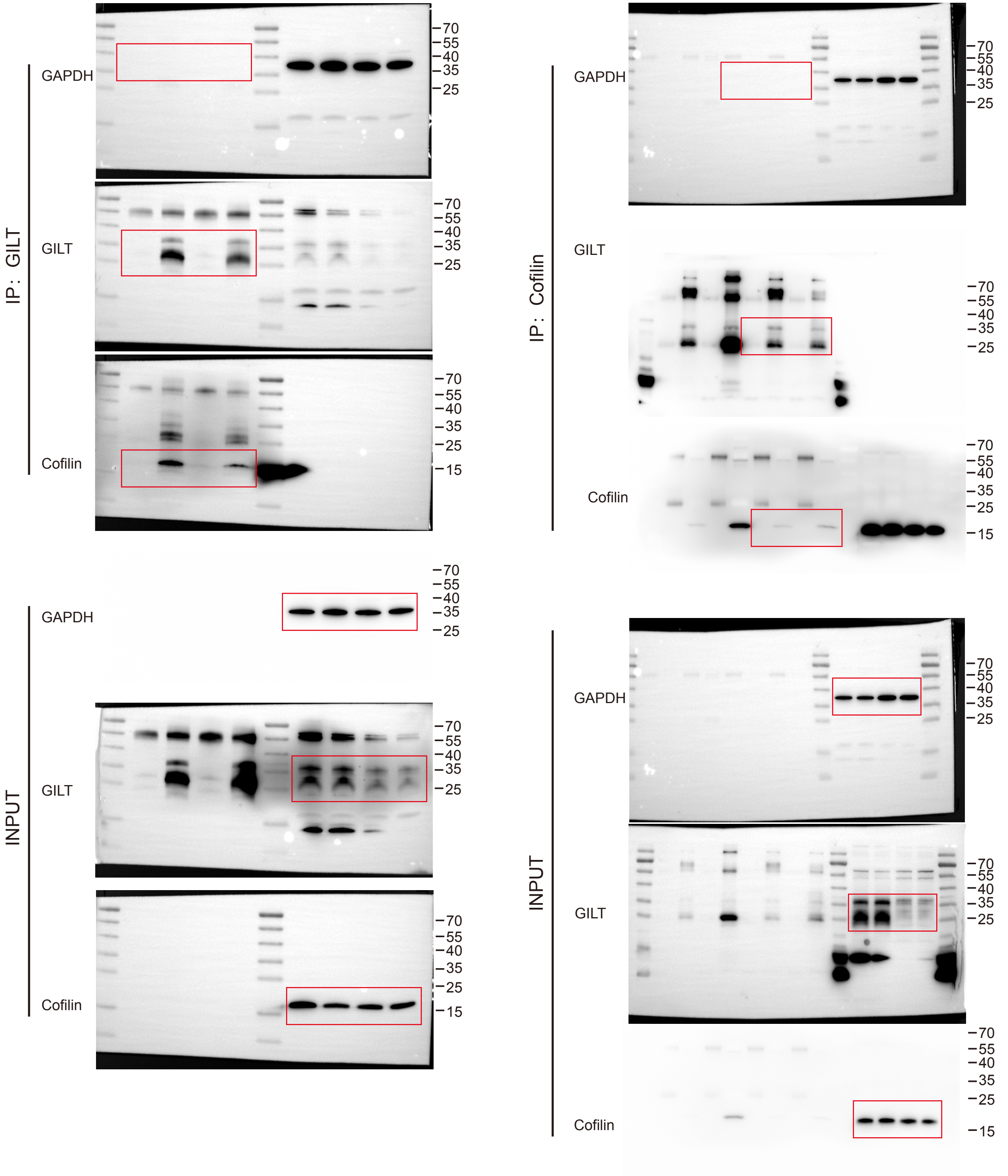
IP：GILT
GAPDH
GILT
Cofilin
INPUT
GAPDH
GILT
Cofilin
IP：Cofilin
GAPDH
GILT
Cofilin
INPUT
GAPDH
GILT
Cofilin
70
55
40
35
25
15

Fig 3G

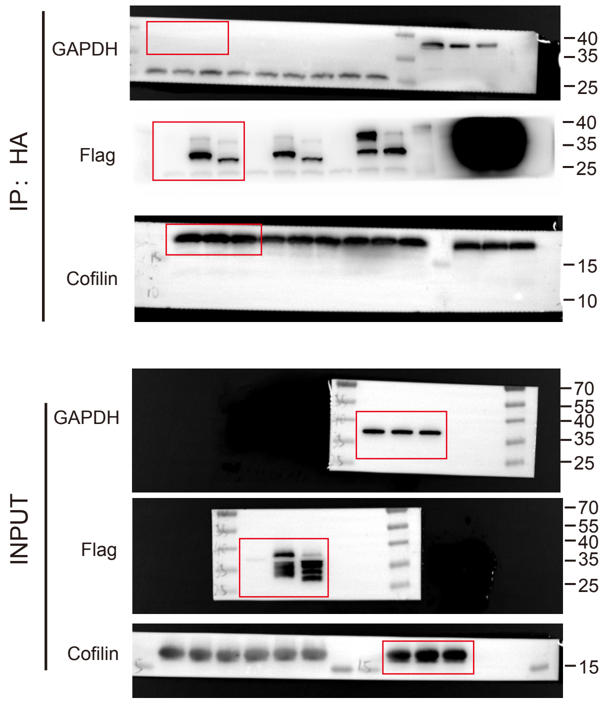
IP：HA
GAPDH
Flag
Cofilin
INPUT
GAPDH
Flag
Cofilin
40
35
25
40
35
25
15
10
70
55
40
35
25
70
55
40
35
25
15

Fig 3H

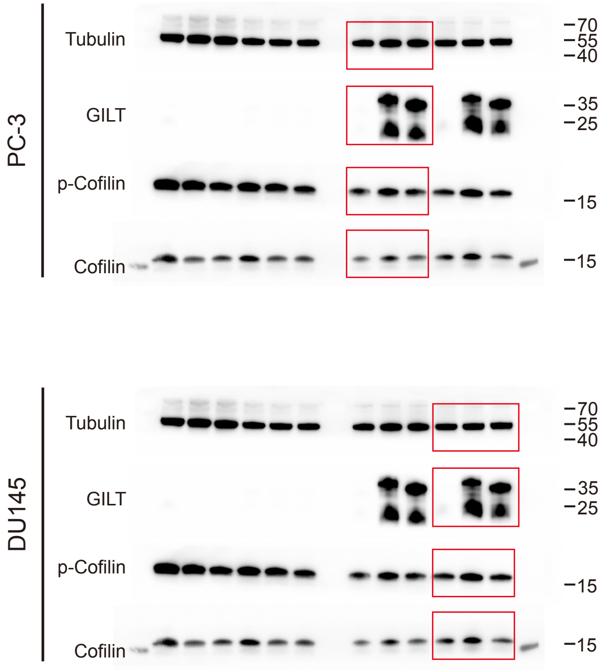
PC-3
Tubulin
GILT
p-Cofilin
Cofilin
DU145
Tubulin
GILT
p-Cofilin
Cofilin
70
55
40
35
25
15
15
70
55
40
35
25
15
15

Fig 4A

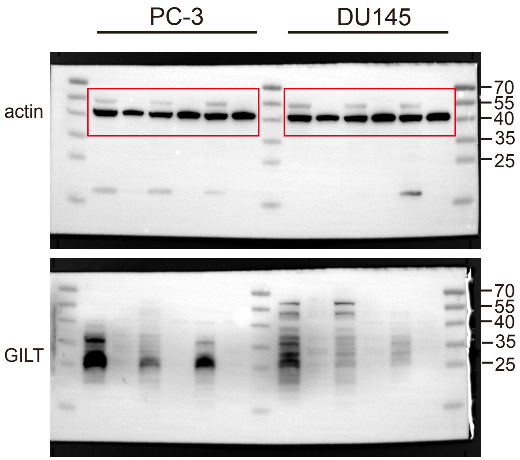
PC-3
DU145
actin
GILT
70
55
40
35
25

Fig 4D

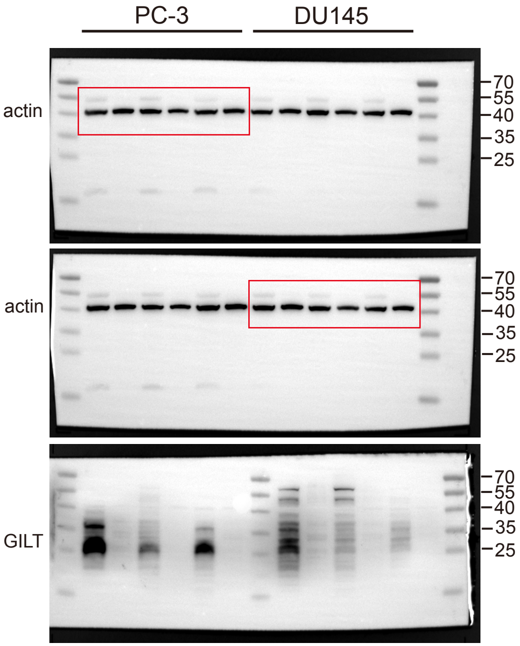
PC-3
DU145
actin
actin
GILT
70
55
40
35
25

Fig 5F

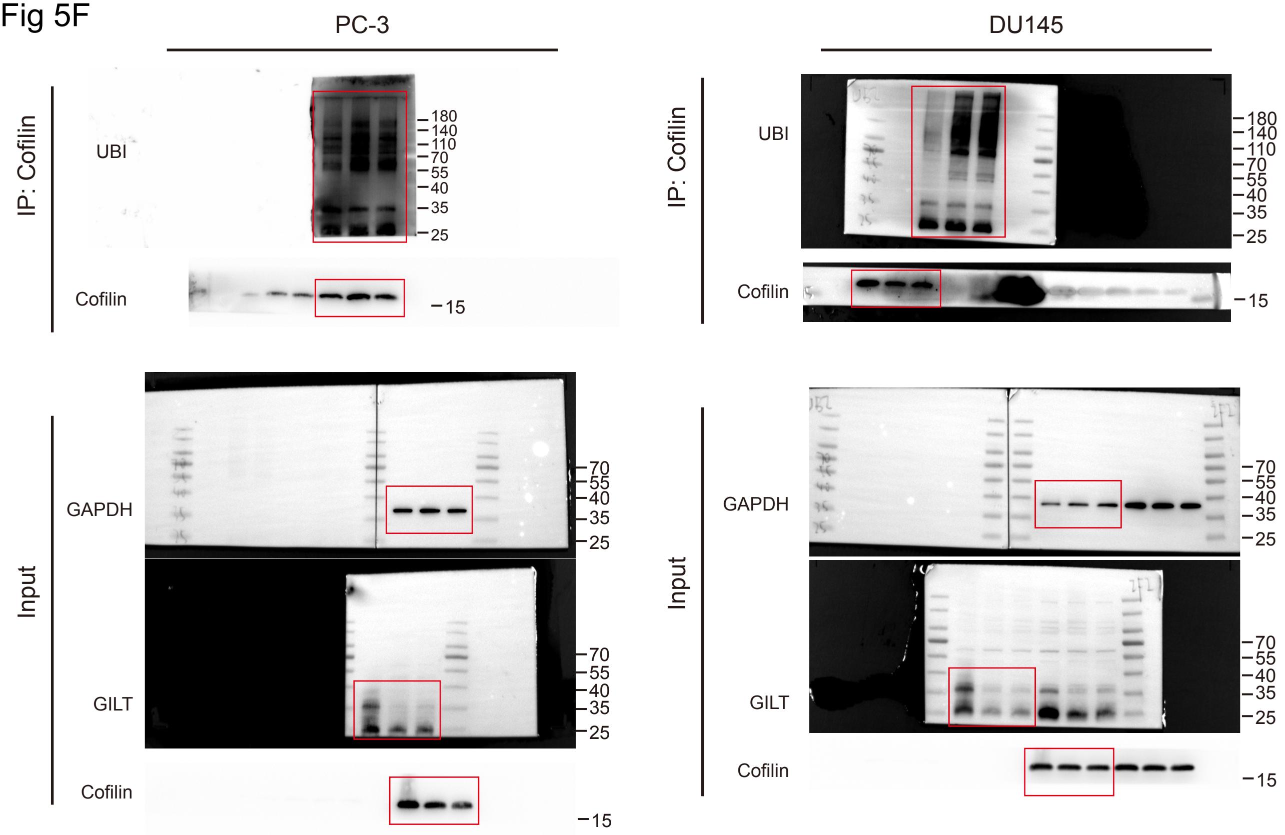

PC-3
DU145
IP: Cofilin
UBI
180
140
110
70
55
40
35
25
Cofilin
15
Input
GAPDH
GILT

Fig 5G

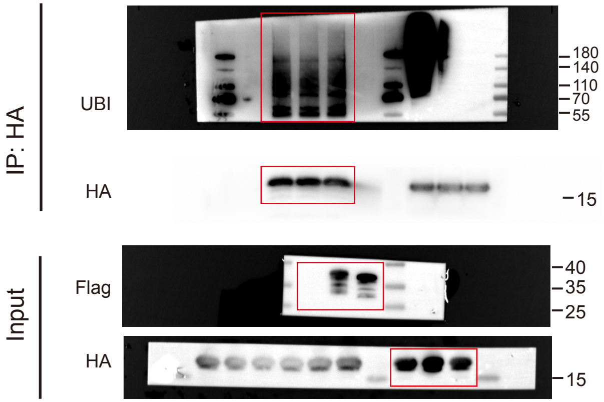

IP: HA
UBI
180
140
110
70
55
HA
15
Input
Flag
40
35
25

Fig 5A

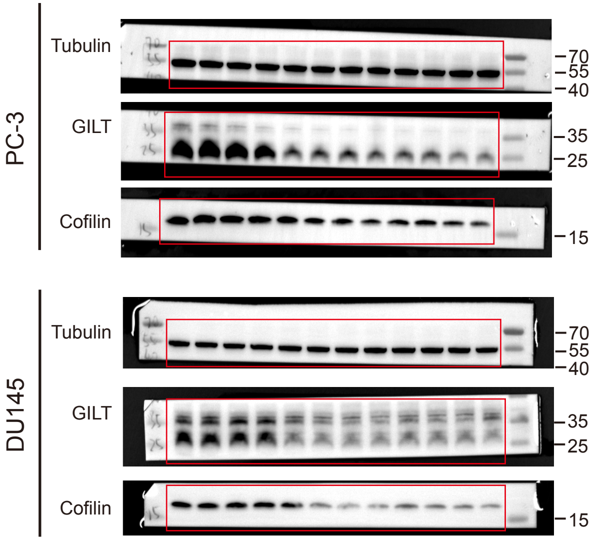

Fig 5B

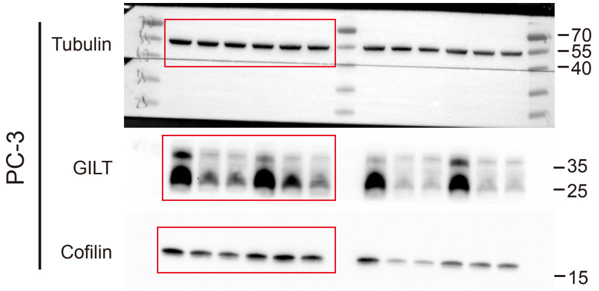

Fig 5C

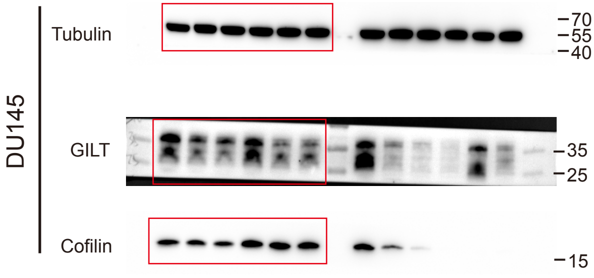

Fig 5D

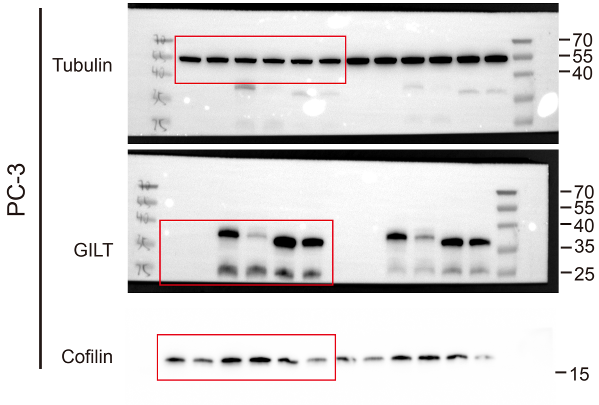

Fig 5E

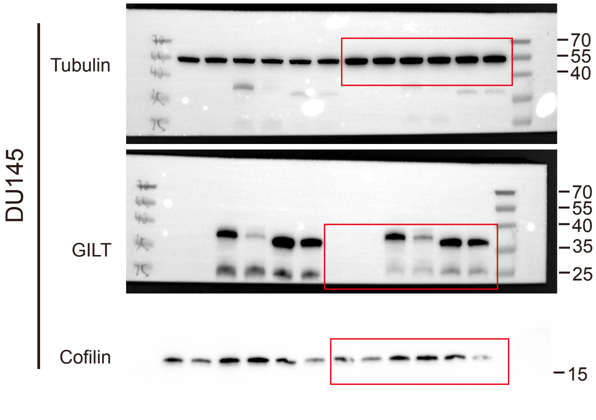

Fig 6A

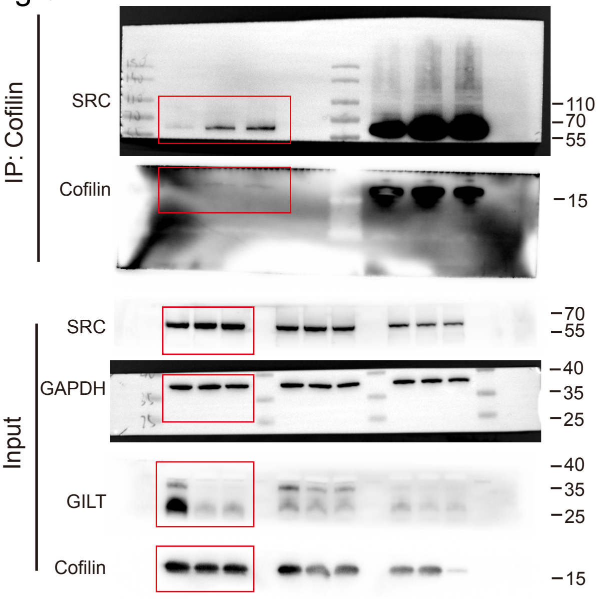

Fig 6B

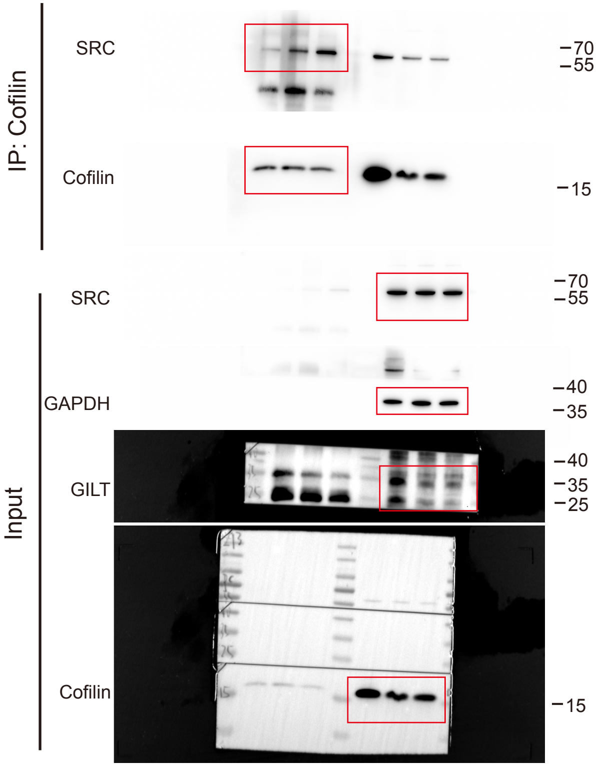

Fig 6C

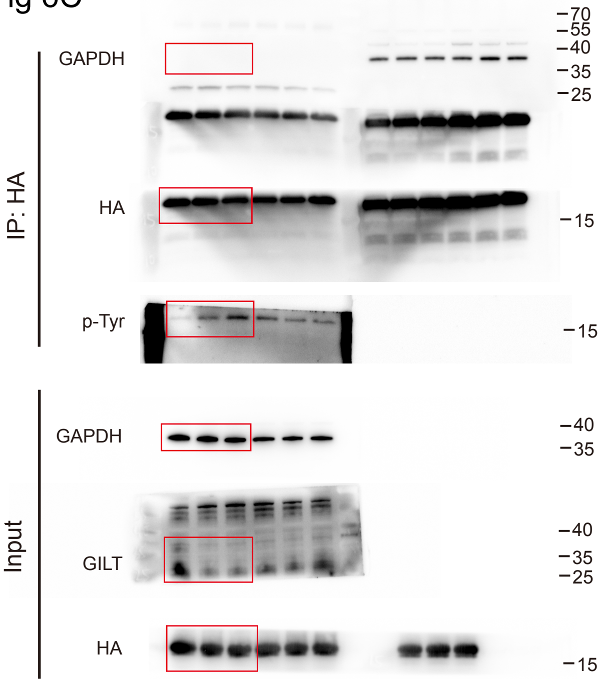

Fig 6D

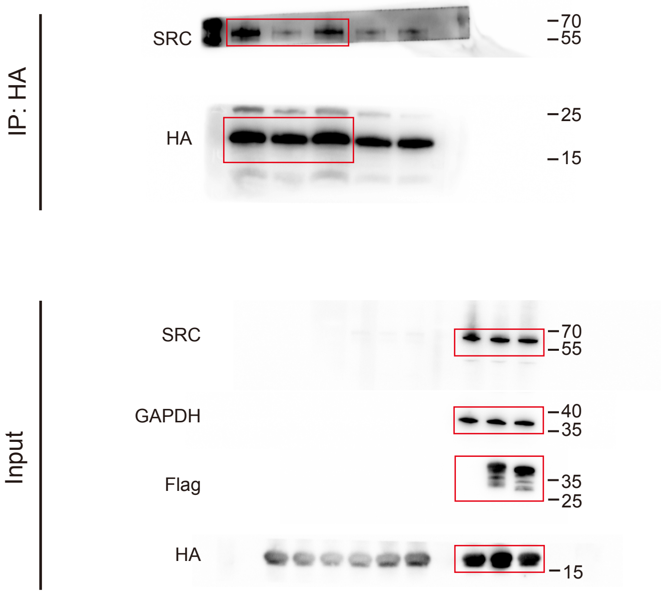

Fig 6E

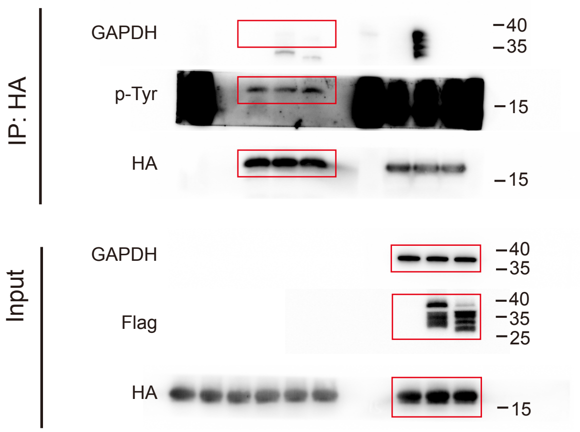

Fig 6F

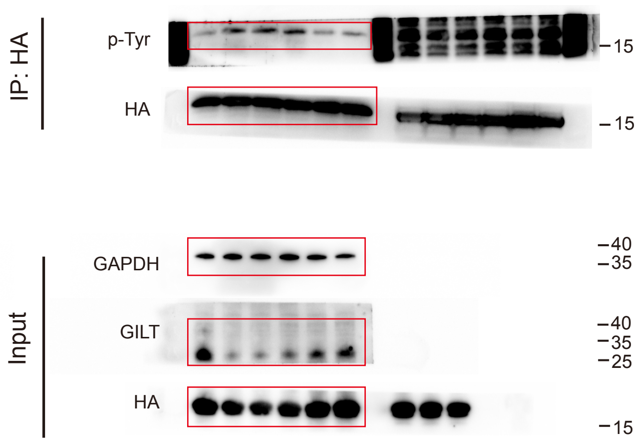

Fig 6G

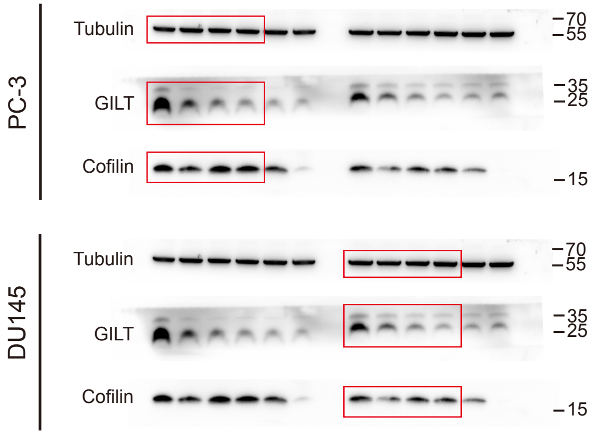

Fig s2B

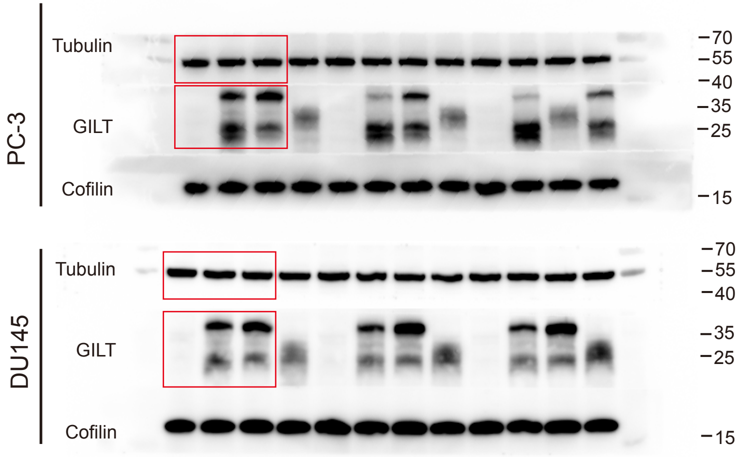

Fig s3C

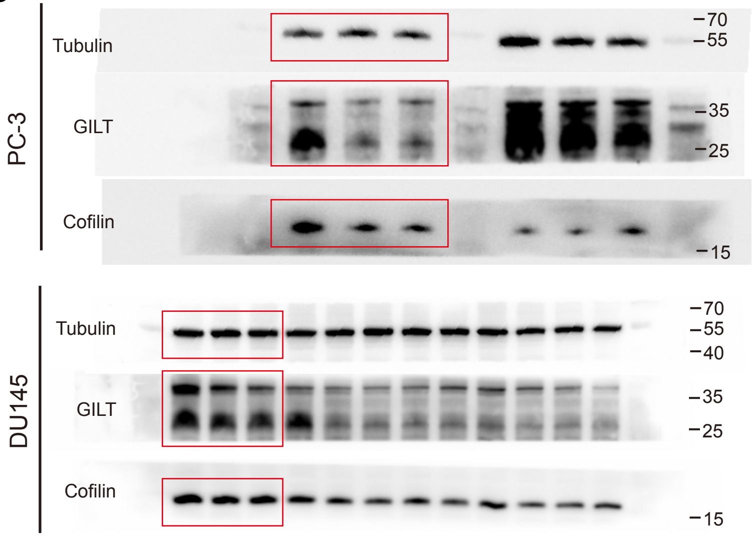

Fig s3E

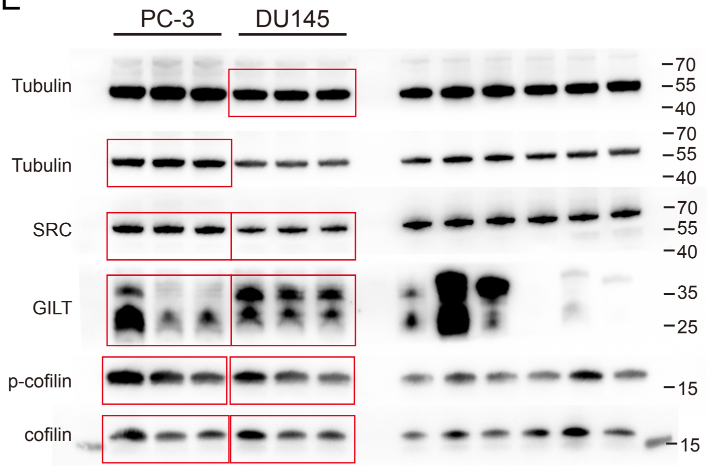

Fig s3G

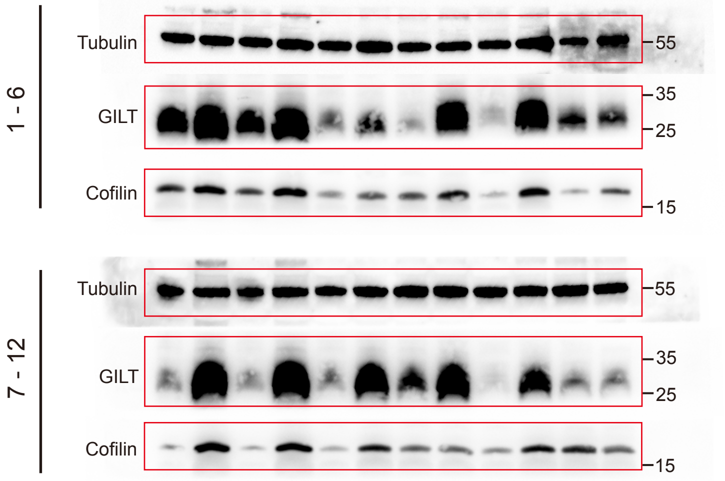

Fig s4A

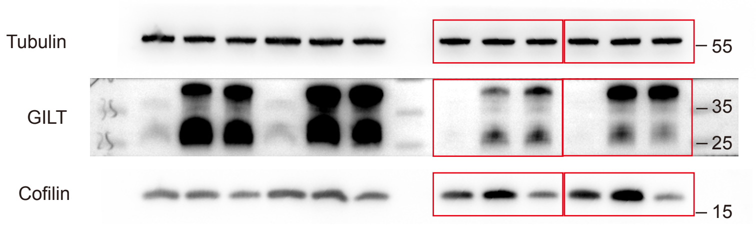

Supplement: Supplementary file 2 — Original full length western blots [file 41420_2025_2288_MOESM2_ESM.pdf]
